# Software Tools for Model-Informed Precision Dosing: How Well Do They Satisfy the Needs?

**DOI:** 10.3389/fphar.2020.00620

**Published:** 2020-05-07

**Authors:** Wannee Kantasiripitak, Ruth Van Daele, Matthias Gijsen, Marc Ferrante, Isabel Spriet, Erwin Dreesen

**Affiliations:** ^1^Therapeutic and Diagnostic Antibodies Unit, Department of Pharmaceutical and Pharmacological Sciences, KU Leuven, Leuven, Belgium; ^2^Clinical Pharmacology and Pharmacotherapy Unit, Department of Pharmaceutical and Pharmacological Sciences, KU Leuven, Leuven, Belgium; ^3^Pharmacy Department, University Hospitals Leuven, Leuven, Belgium; ^4^Department of Gastroenterology and Hepatology, University Hospitals Leuven, Leuven, Belgium; ^5^Translational Research Center for Gastrointestinal Disorders, Department of Chronic Diseases, Metabolism and Ageing, KU Leuven, Leuven, Belgium

**Keywords:** model-informed precision dosing, therapeutic drug monitoring, target concentration intervention, software tool, pharmacometrics

## Abstract

Model-informed precision dosing (MIPD) software tools are used to optimize dosage regimens in individual patients, aiming to achieve drug exposure targets associated with desirable clinical outcomes. Over the last few decades, numerous MIPD software tools have been developed. However, they have still not been widely integrated into clinical practice. This study focuses on identifying the requirements for and evaluating the performance of the currently available MIPD software tools. First, a total of 22 experts in the field of precision dosing completed a web survey to assess the importance (from 0; do not agree at all, to 10; completely agree) of 103 pre-established software tool criteria organized in eight categories: user-friendliness and utilization, user support, computational aspects, population models, quality and validation, output generation, privacy and data security, and cost. Category mean ± pooled standard deviation importance scores ranged from 7.2 ± 2.1 (user-friendliness and utilization) to 8.5 ± 1.8 (privacy and data security). The relative importance score of each criterion within a category was used as a weighting factor in the subsequent evaluation of the software tools. Ten software tools were identified through literature and internet searches: four software tools were provided by companies (DoseMeRx, InsightRX Nova, MwPharm++, and PrecisePK) and six were provided by non-company owners (AutoKinetics, BestDose, ID-ODS, NextDose, TDMx, and Tucuxi). All software tools performed well in all categories, although there were differences in terms of in-built software features, user interface design, the number of drug modules and populations, user support, quality control, and cost. Therefore, the choice for a certain software tool should be made based on these differences and personal preferences. However, there are still improvements to be made in terms of electronic health record integration, standardization of software and model validation strategies, and prospective evidence for the software tools’ clinical and cost benefits.

## Introduction

“First, do no harm” is a fundamental dictum in pharmacotherapy. Nevertheless, we may want to raise the bar and aim for optimal efficacy with minimal toxicity in all patients. Although this may seem evident, therapeutic failure and toxicity are still very frequent in clinical practice. The standard label-recommended dosing regimens may not be effective and safe in all patients due to large interpatient variability in exposure and response. To improve drug treatment outcomes and avoid adverse drug reactions in individual patients, a precision dosing approach has been proposed, which aims at the precise attainment of predefined drug exposure targets (Polasek et al., 2019a). The precision dosing approach is justified when pharmacokinetic (PK) variability exceeds the limits of a safe and effective range of drug exposure (Holford and Buclin, 2012). Since inter- and intra-patient PK variability can be quantified and taken into account by employing population PK models, such models can be used to predict the optimal dose of a drug in an individual patient (Polasek et al., 2018). This model-based approach has been referred to as model-informed precision dosing (MIPD) in recent publications (Darwich et al., 2017; Polasek et al., 2019c).

MIPD involves the application of mathematical and statistical algorithms using simultaneous integration of patient covariates (*i.e.*, *a priori* prediction) and individual drug concentration measurements (*i.e.*, *a posteriori* prediction or Bayesian forecasting). Therefore, MIPD is often perceived as a complicated and time-consuming task. To overcome these obstacles, these models have been implemented in software tools to support clinical decision-making on therapeutic individualization. The first computer-based algorithms for dose prediction were introduced half a century ago (Jelliffe, 1969; Sheiner, 1969; Jelliffe et al., 1972; Sheiner et al., 1972). However, fifty years later, apart from some isolated local efforts (Barrett, 2015; Van der Zanden et al., 2017), MIPD has not been widely implemented in routine clinical practice.

Barriers that hampered MIPD software tools from being widely implemented in health care include little published evidence of large-scale utility and impact of these software tools, lack of user-friendliness, lack of technical expertise at practice site, and cumbersome validation of the software tools in clinical settings (Darwich et al., 2017). To ensure wider integration of MIPD software tools in routine clinical use, the software tool functionalities should align with the requirements of the end-users (*i.e.*, healthcare professionals) (Darwich et al., 2017). In the past few years, MIPD has gained renewed attention as a result of the increasing awareness that one dose does not fit the needs of all patients, especially in special populations, such as frail elderly patients, pediatric patients, patients with renal or hepatic impairment, and critically ill patients (Kohn et al., 2014; Collins and Varmus, 2015). This renewed attention is evidenced by the publication of opinion papers, the scheduling of various dedicated conference sessions (ASCPT, PAGE, ACoP, and ACCP), the creation of a special interest group within ISoP (“Applied Clinical Pharmacometrics”), and most importantly the release of new MIPD software tools (Keizer et al., 2018).

Hence, evaluation of the current status of MIPD software tools and comparison with previous conclusions on this topic is needed. Therefore, we aimed to (i) identify requirements that MIPD software tools should comply with based on experts’ opinions, and to (ii) compare performances of the currently available MIPD software tools based on these requirements. This information can assist health care professionals in selecting the software tool that fits best their specific needs.

## Materials and Methods

### Search Strategy and Selection Criteria

MIPD software tools were identified through searching PubMed, Google, Google Scholar, Web of Science, and the Population Approach Group in Europe (PAGE) website until February 2020 by using the following Medical Subject Headings (MeSH) terms and free text variations of these terms: “software”, “software tool”, “dosing software”, “dashboard”, “precision dosing”, “model-informed precision dosing”, “model-based precision dosing”, “therapeutic drug monitoring”, “target concentration intervention”, “adaptive feedback control”, “concentration control”, and “Bayesian”. These terms were combined with Boolean logical operators “and” and “or”. Reference lists were hand-searched for other relevant literature. The MIPD software tools identified through these searches had to meet the following selection criteria: (i) the software is available and actively updated, (ii) the software has a graphical user interface (GUI), (iii) the software is capable of Bayesian forecasting, (iv) the software supports more than one drug module, and (v) the software provider accepts participation in this study.

### Establishing Evaluation Criteria

The criteria used to benchmark the MIPD software tools were defined based on a literature review and the experts’ opinion (see *Experts’ Opinion*). These evaluation criteria were grouped into eight categories related to (i) user-friendliness and utilization, (ii) user support, (iii) computational aspects, (iv) population models, (v) quality and validation, (vi) output generation, (vii) privacy and data security, and (viii) cost (**Supplementary Table 1**). For criteria with binary classification (yes/no), a score of either 0 or 1 was assigned with 1 indicating the best performance. For ordered categorical criteria with <10 categories, a score of 0 to 1 with stepsize 1/(*n*-1) was assigned with the highest score indicating the best performance. For continuous criteria (*i.e.*, ordered categorical with ≥10 categories), a score ranging from 0 (for the lowest performance) to 1 (for the highest performance) with stepsize 0.1 was assigned. NA was assigned when not applicable.

### Experts’ Opinion

Clinicians, pharmacists, and pharmacometricians active in the field of precision dosing were invited to participate in a web survey that queried about the level of importance of each of the established evaluation criteria that were used to evaluate the software tools (**Supplementary Data 1**). The criteria were scored on a scale from 0 to 10, with 0 indicating “I do not agree at all that this criterion is important” and 10 indicating “I completely agree that this criterion is important.” There was also an “undecided” option for every question indicating “I think that my level of knowledge is not sufficient to evaluate this criterion.” Moreover, experts could suggest additional criteria regarding each category. The scores representing the levels of importance were then used as weighting factors in the benchmarking to calculate the final score for each evaluation criterion.

### Software Tool Evaluation

The selected MIPD software tools were independently evaluated by four authors (WK, RVD, MG, and ED) using the established evaluation criteria. Benchmarking scores were calculated based on an evaluation grid consisting of the scoring definitions and the possible scores of each criterion (**Supplementary Table 1**). Standalone versions of the software tools were evaluated. The evaluations were performed on one desktop and three laptop computers with 64-bit operating system Windows 10 Enterprise. The web-based software tools were accessed through the Google Chrome web browser.

Next to the evaluation by the authors, some criteria were evaluated based on the software provider’s answers in a web survey (**Supplementary Data 2**). A web survey was filled out by all of the software providers. This web survey consisted of two parts. The first part of the survey queried the descriptive characteristics of the MIPD software tool. The second part queried features of the MIPD software tool over the eight aforementioned categories. To facilitate the benchmarking, a maximum 1-hour online introduction was allowed upon request of the software providers to obtain more information. Also, the benchmarking scores of the criteria that were evaluated based on the software provider’s answers were cross-checked by the providers to allow a double-control and confirmation.

### Data Analysis

Data were imported in R (version 3.6.1; R Foundation for Statistical Computing, R Core Team, Vienna, Austria) for data wrangling, visualization, and statistical analyses. Graphics were generated using the ggplot2 package in RStudio (version 1.2.5001; R Studio, Inc., RStudio Team, Boston, MA, USA). Descriptive statistics were stated as percentages for discrete variables and as mean ± standard deviation (SD) or median (minimum-maximum) for continuous variables.

The scores of the experts’ opinions on the importance of each criterion were summarized by category using the within-category mean and the pooled within-category standard deviation (*S*_pooled_;

Eq. 1 $$S_{\text{pooled}}=\sqrt{\frac{(n_{1}-1)s_{1}^{2}\text{+(}n_{2}-1)s_{2}^{2}\text{+…+(}n_{k}-1)s_{k}^{2}}{n_{1}+n_{2}+\ldots +n_{k}-k}},$$

with *s* the standard deviation of each criterion, *n* the number of responses in each criterion, and *k* the number of criteria within the category).

The average scores of the experts’ opinion on the importance of each criterion were used to compute the weighting factors. The relative weighting factor *w_rel_* for criterion *i* was calculated by dividing the average score assigned to this criterion *w_i_* by the sum of the average scores of all criteria in that category *k*;

Eq. 2 $$w_{\mathrm{rel},i}=\;\frac{W_{i}}{\Sigma_{i=1}^{k}W_{i}},$$

to normalize the sum of the relative weighting factors in each category to 1. The relative weighting factor for each criterion was multiplied with the benchmarking score given to that criterion, to obtain importance-weighted benchmarking scores. The importance-weighted benchmarking scores were summed by category and compared between the MIPD software tools. In addition, a ranking of the MIPD software tools was established by summing the importance-weighted benchmarking scores to obtain an overall performance score for each evaluated MIPD software tool.

## Results

### Included Software Tools

**Figure 1:**
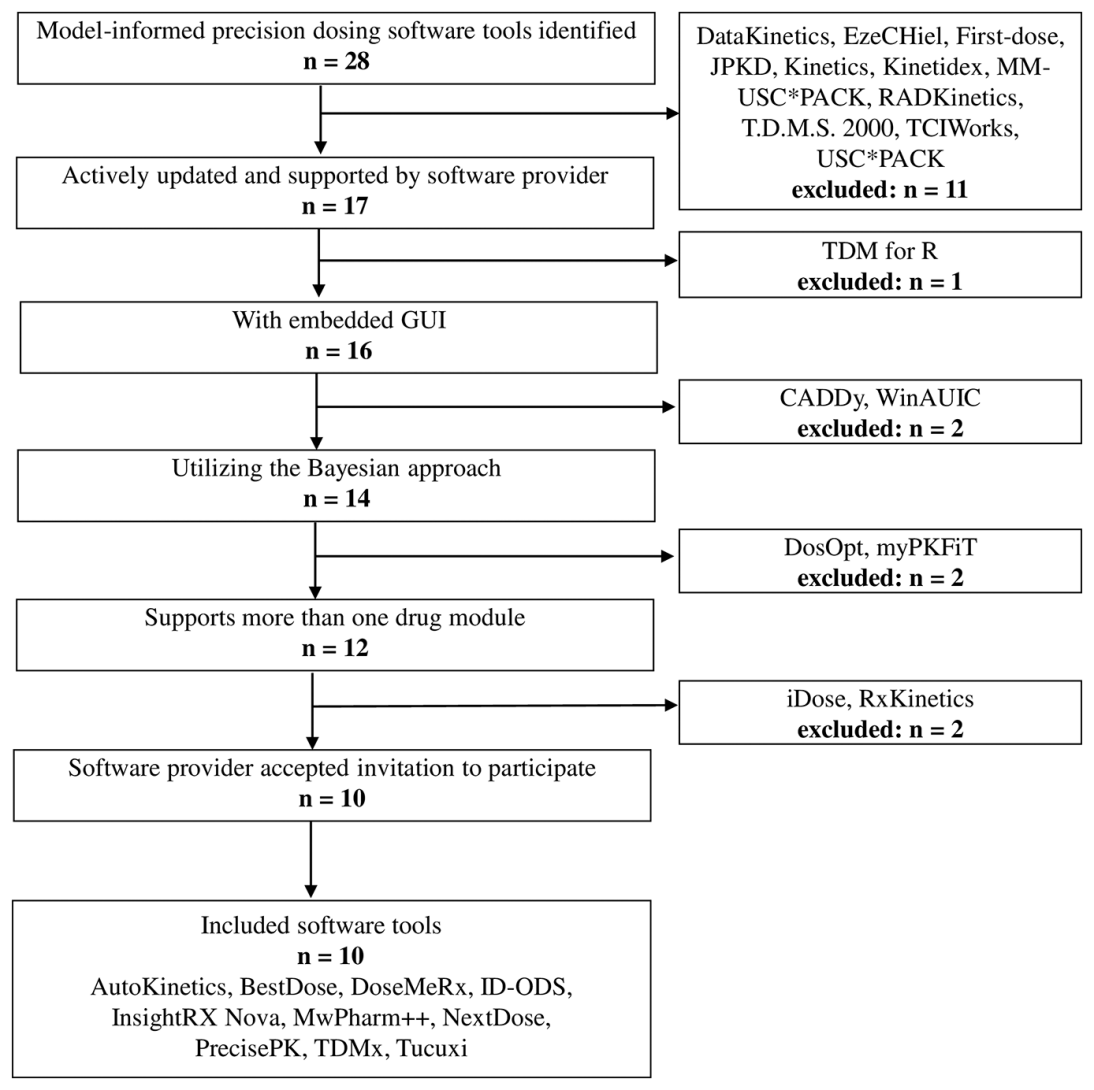
Flowchart of the included and excluded model-informed precision dosing software tools. GUI, graphical user interface.

Twenty-eight MIPD software tools were identified, of which 10 were included in this study (**Figure 1**). Two software providers (iDose and RxKinetics) did not accept participation in our study. The provider of iDose declined participation due to an ongoing update. For RxKinetics, we did not receive a response from the provider. Descriptive characteristics of the included MIPD software tools are presented in **Table 1**. The earliest release year amongst the included software tools was 2012 (NextDose). Four out of 10 software tools are provided by software companies (DoseMeRx, InsightRX Nova, MwPharm++, and PrecisePK). The others are non-company providers (AutoKinetics, BestDose, ID-ODS, NextDose, TDMx, and Tucuxi). Seven out of 10 software tools serve both research and clinical purposes. While BestDose only serves a research purpose, ID-ODS and Tucuxi only serve a clinical purpose. All of the evaluated software tools have a web-based version available, except MwPharm++ and Tucuxi. All of the evaluated software tools have a standalone version except AutoKinetics.

**Table 1:** Descriptive characteristics of the model-informed precision dosing software tools.

	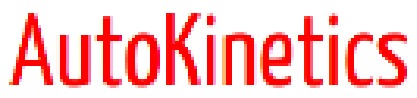 AutoKinetics	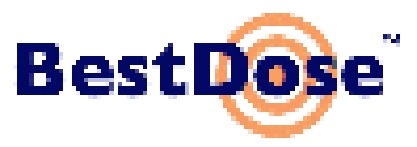 BestDose	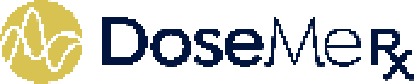 DoseMeRx	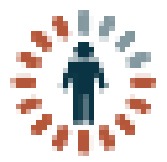 ID-ODS	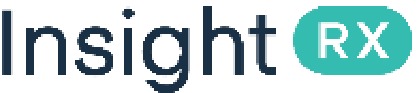 InsightRX Nova	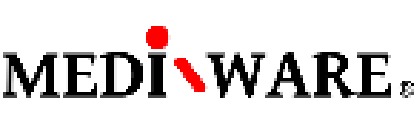 MwPharm++	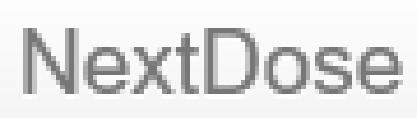 NextDose	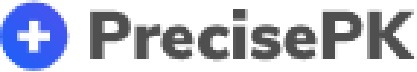 PrecisePK	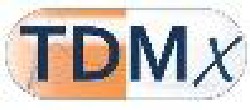 TDMx	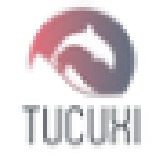 Tucuxi
*Founder*	Paul Elbers Rob Bosman	Roger Jelliffe	Robert McLeay	Andras Farkas Gergely Daroczi	Sirj Goswami Ron Keizer Ranvir Mangat	Johannes H. Proost Cees Neef Jiří Potůček Nieko Punt	Sam Holford Nick Holford	Philip Anderson Anjum Gupta	Sebastian Wicha	Yann Thoma
*CEO*	NA	NA	Charles Cornish	NA	Sirj Goswami	Jiří Potůček	NA	Anjum Gupta	NA	NA
*Company/* *institution*	Departments of Intensive Care Medicine of Amsterdam UMC, location VUmc and OLVG Oost Hospital	Laboratory of Applied Pharmacokinetics and Bioinformatics, Children’s Hospital Los Angeles	DoseMe (Tabula Rasa HealthCare Company)	Optimum Dosing Strategies	Insight Rx Inc.	Mediware a.s.	University of Auckland	Healthware Inc.	Institute of Pharmacy, University of Hamburg	School of Engineering and Management Vaud (HEIG-VD)
*Location of company/* *institution*	Amsterdam, The Netherlands	Los Angeles, California, USA	Moorestown, New Jersey, USA	Bloomingdale, New Jersey, USA	San Francisco, California, USA	Groningen, The Netherlands/Prague, Czech Republic	Auckland, New Zealand	San Diego, California, USA	Hamburg, Germany	Yverdon-les-Bains, Switzerland
*Previous version names*	–	MM-USC*PACK	–	–	InsightRX Software	MwPharm DOS, MwPharm 4.0	–	T.D.M.S.	–	–
*Release date of the first version*	1 August 2018 (desktop) 1 March 2018 (web-based)	1 October 2018	11 July 2014	23 August 2013 (web-based) 23 August 2013 (mobile application)	1 June 2015	1 January 2015 (desktop)	1 April 2012	1 January 1986 (desktop) 11 November 2019 (web-based)	1 January 2015	1 June 2017
*Reviewed version*	Web-based version 1.2.0	Web-based version 0.2.0	Web-based version 2.11.13	Web-based version 2.9.1-20191010.d4baf19	Web-based version 1.16.1	Desktop version 1.7.5	Web-based version 1.6.0	Web-based version 19.07.26	Web-based version Beta	Desktop version Gui Git revision: 60435e8ee, Tucucore Git revision: 33280802
*Computer language of source code*	Asp.net and vb.net	Fortran, R	Perl, R, python	Ionic, R	R, JavaScript	C#	Javascript, PHP, MySQL, NM-TRAN	C++, PHP Web App: JSX, C++	R/C++	C++
*Software version* *(compatible platform or mobile application name or website)*	Desktop (Windows), Web-based	Desktop (Windows), Web-based (bestdoserx.com/)	Web-based (app.doseme-rx.com), Android and iOS (DoseMe)	Web-based (app.id-ods.org), Android (ID-ODS Adult), iOS (app.id-ods.org)	Web-based (pk.insight-rx.com)	Desktop (Windows), Web-based, Android, iOS (mwpharm.online)	Web-based (nextdose.org)	Desktop (Windows, Mac), Web-based (app.precisepk.com/login)	Web-based (tdmx.eu/Launch-TDMx/)	Desktop (Windows, Mac, Linux)
*Website*	autokinetics.eu	lapk.org/bestdose.php	doseme-rx.com	optimum-dosing-strategies.org/id-ods/	insight-rx.com	mediware.cz	nextdose.org	precisepk.com	tdmx.eu/	tucuxi.ch
*Purpose of use*	research and clinical	research	research and clinical	clinical	research and clinical	research and clinical	research and clinical	research and clinical	research and clinical	clinical

*NA, not applicable because not a company.

### Experts’ Opinion

**Figure 2:**
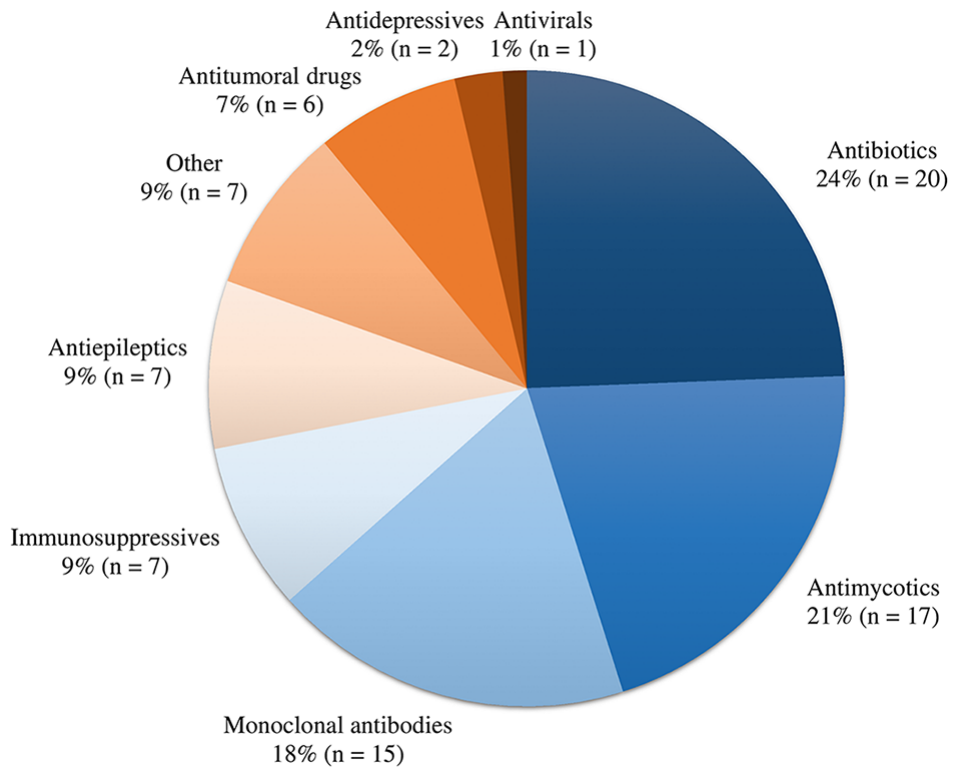
Overview of drug classes involved in precision dosing programs of the participating experts.

**Figure 3:**
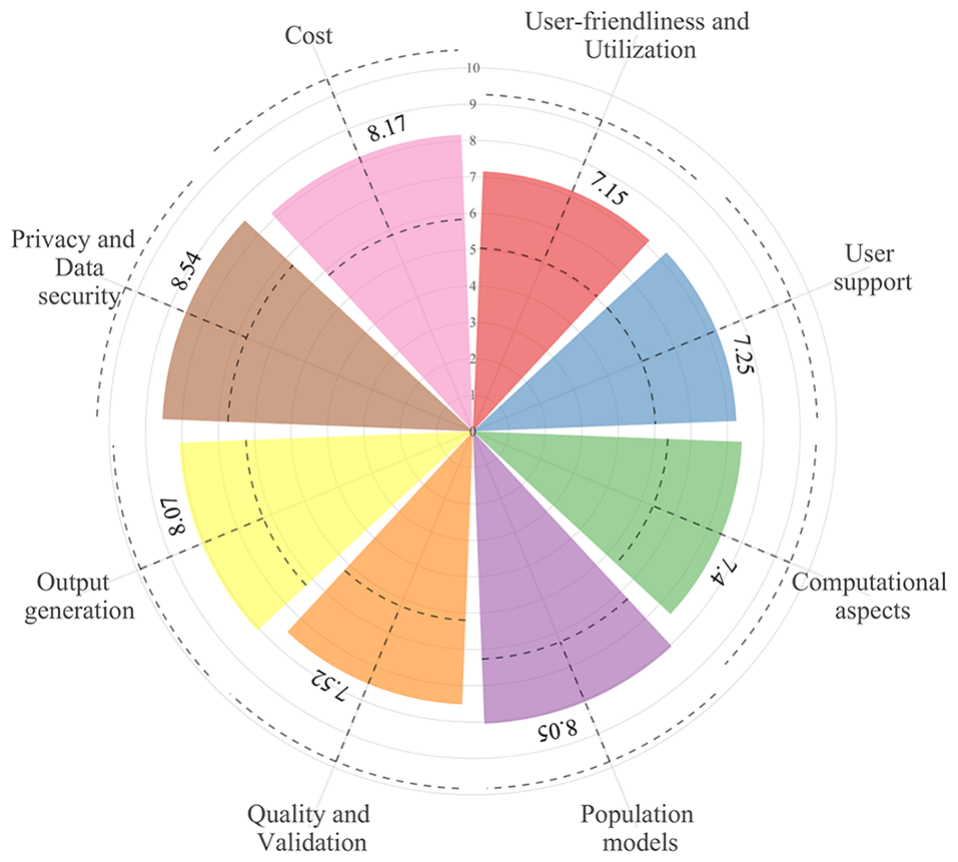
The overall mean (±1 pooled standard deviation; dashed lines) of importance levels of the considered criteria in the eight categories.

A total of 22 out of 63 (35%) contacted experts (seven clinicians, six pharmacists, and nine pharmacometricians) have completed the survey (**Supplementary Data 1**). Fifteen of them indicated to be involved in precision dosing programs at least weekly, mostly in the domain of antimicrobials and monoclonal antibodies (**Figure 2**). The mean ± pooled SD of the importance levels ranged from 7.15 ± 2.11 (user-friendliness and utilization) to 8.54 ± 1.80 (privacy and data security) as illustrated in **Figure 3**. Distributions of scores of experts’ opinions in each criterion by category are reported in **Supplementary Figures 1–8**. The six criteria evaluated as most important, with an average score above nine, were (i) the software should be able to propose *a priori* and *a posteriori* dosing regimens, (ii) the software should provide models developed in relevant populations, (iii) suitable diagnostic tools and/or methods should be used in model selection prior to implementing a model in the software, (iv) the model qualification should be performed for “fit for purpose” prior to software, (v) the dosing recommendation from the software should be straightforward and easy to understand, and (vi) software should comply with the European Union General Data Protection Regulation (EU GDPR) or equivalent. The least important criterion, with an average score below five, was the pharmaceutical industry should have been involved in software development. Moreover, experts did not suggest additional evaluation criteria in addition to the already established ones.

### Benchmarking

**Figure 4:**
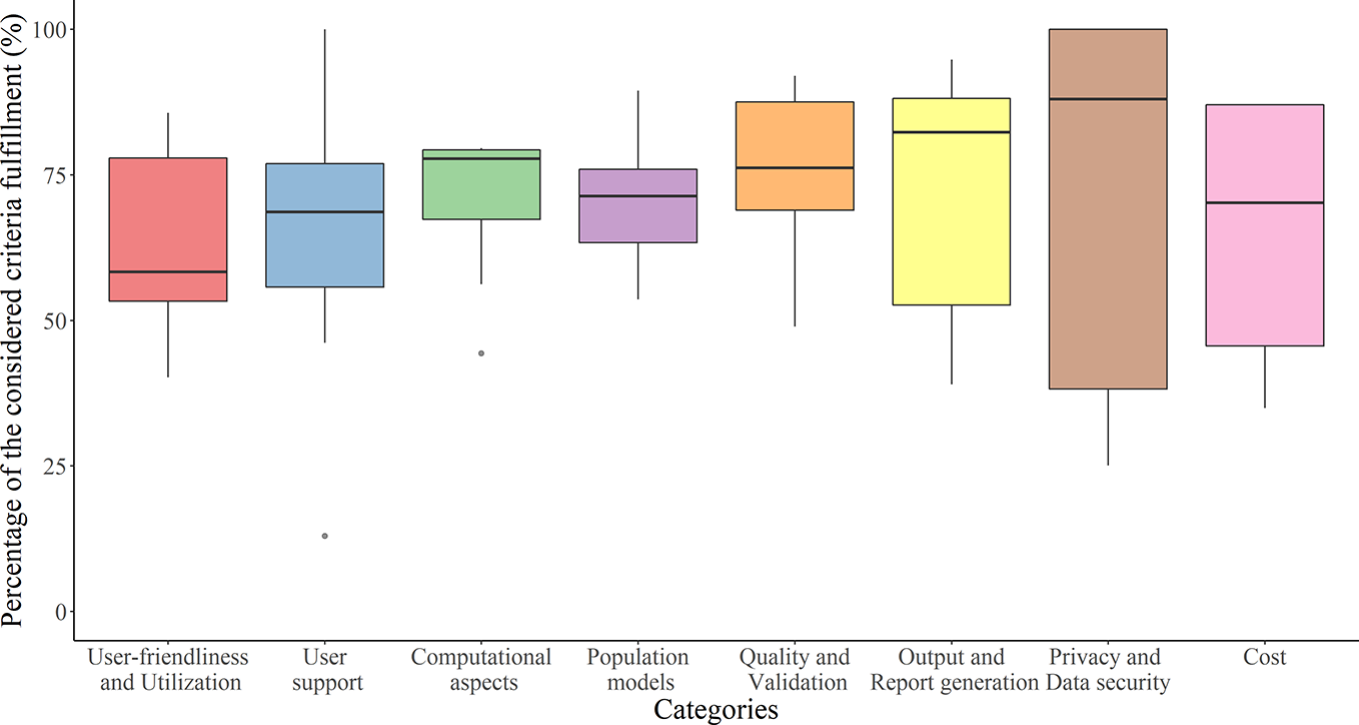
Tukey boxplot representing fulfillment of the considered criteria by the 10 evaluated software tools in each category.

**Figure 5:**
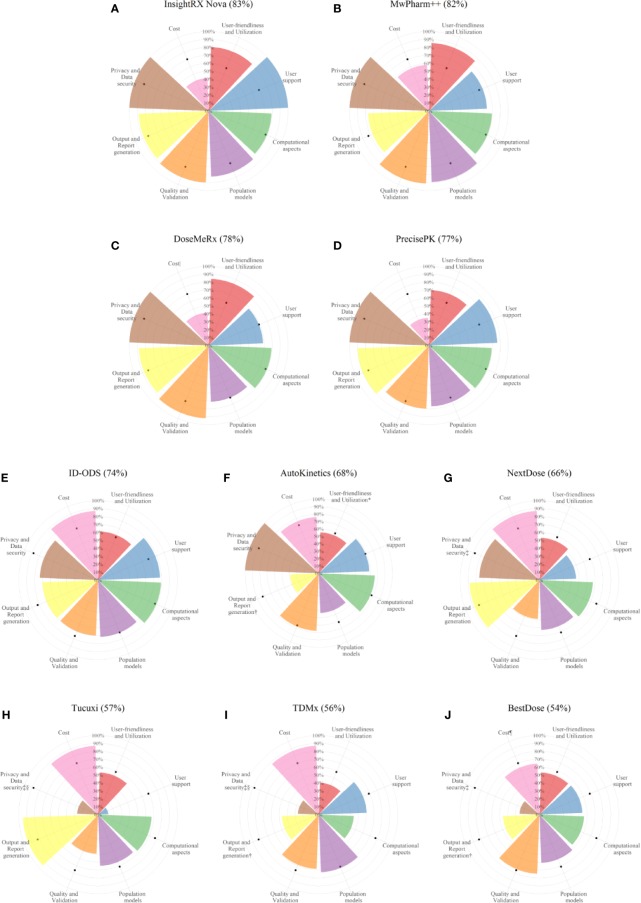
Fulfillment of the considered criteria in the eight categories by each of the evaluated software tools. Numbers in parentheses are percentage of the overall performance scores. Software tools are ranked in decreasing order of overall performance scores [from the highest score **(A)** to the lowest score **(J)**]. Black solid circles in each category represent the median fulfillment (%) of the considered criteria by the 10 evaluated software tools. ^*^Manual data entry not possible. ^†^A report cannot be generated. ^‡^The data privacy method in data collection cannot be evaluated since no data are collected in the software. ^§^Database encoding cannot be evaluated since no data are stored in the software. ^|^An individual license is not available. ^¶^An institution license is not available.

Benchmarking scores of the evaluated software tools with the relative weighting factor of each criterion are reported in **Supplementary Table 2**. The distribution of the percentage of the fulfilled requirements by category is reported in **Figure 4**. The overall performance of each software tool and the percentage of the fulfilled requirements in each category are illustrated for every evaluated software tools in **Figure 5**.

### User-Friendliness and Utilization

The evaluated software tools fulfilled user-friendliness and utilization criteria for 58% (40–86%). MwPharm++ (86%), DoseMeRx (84%), and InsightRX Nova (81%) fulfilled the considered criteria the most. Software tools differed most in terms of easiness in manual data entry and the capability of electronic health record (EHR) integration.

Most software tools are available as a web-based software apart from Tucuxi which is only available as a desktop software. The desktop software can be downloaded *via* the software websites. For TDMx, users can freely access its web-based software tools without registration required. Six software tools can be integrated into the EHR (AutoKinetics, DoseMeRx, InsightRX Nova, MwPharm++, PrecisePK, and Tucuxi). The installation of EHR-integrated version may require technical support. In addition, ID-ODS is currently in the process of integrating its software tools with the EHR.

All software tools with input data storage capability provide database search by patient name, patient identification, drug name, or date. The benchmarking score of easiness in manually data entry criterion was highest in DoseMeRx, InsightRX Nova, and PrecisePK. These software tools provide structured layout and toolbox widgets that assist users in entering data. In addition, they scored highest on global visual appeal.

### User Support

The evaluated software tools fulfilled the user support criteria for 69% (13–100%). InsightRX Nova (100%), PrecisePK (86%), and ID-ODS (78%) fulfilled the considered criteria the most. Differences between software tools are mostly explained by the type of user support services and availability of an online discussion forum for the software users.

Most of the software providers offer both on-site and online user training, except NextDose and Tucuxi. All software tools provide support documentation (*i.e.*, a clinical manual or a technical manual) to the user, except Tucuxi and BestDose. In addition to the user manuals, 24/7 user support as a helpdesk (AutoKinetics), a call support (PrecisePK), and web support services and live chat (DoseMeRx and InsightRx Nova) are provided to the users. InsightRX Nova and BestDose also host a discussion forum for online support.

### Computational Aspects

The evaluated software tools fulfilled the computational aspects criteria for 78% (44–80%). MwPharm++ (80%), DoseMeRx (79%), InsightRX Nova (79%), PrecisePK (79%), and ID-ODS (79%) fulfilled the considered criteria the most.

All software tools require a maximum of four gigabytes of random access memory for running the software (common in computers these days). Only MwPharm++ requires.NET Framework 4.0 to run the software. None of the web-based software tools can access to previous versions, except for AutoKinetics. However, lists of changes and bug fixes between versions have been documented for all web-based software tools except Autokinetics and TDMx. Although Tucuxi is a desktop software, its previous versions are not accessible and there are no changes documented. None of the software tools provide their source code to the user, except for AutoKinetics that will share part of its source code publicly after completing clinical trial. All of the software tools provide error or warning messages to the users when unusual results are obtained. Moreover, structured data can be imported into all company-provided software tools, ID-ODS, and Tucuxi.

### Population Models

The evaluated software tools fulfilled the criteria related to population models for 71% (54–89%). MwPharm++ (89%), InsightRX Nova (83%), and PrecisePK (77%) were the three software tools that fulfilled the considered criteria the most.

Differences between software tools are mostly explained by the number of included drugs and population models. The number of drugs covered by a software tool varies from five in AutoKinetics, BestDose, and TDMx to more than 180 in MwPharm++. Antibiotics are included in all of the evaluated software tools. The two most included antibiotics are vancomycin (9/10 software tools, excluding TDMx) and gentamicin (8/10 software tools, excluding AutoKinetics and BestDose). There are three software tools that only support antibiotics (AutoKinetics, ID-ODS, TDMx). However, monoclonal antibodies, for which there is an emerging interest in precision dosing (Dreesen et al., 2017), have only been included in two of the evaluated software tools (infliximab in MwPharm++ and InsightRX Nova, adalimumab in InsightRX Nova). In addition to various drug classes, company software tools provide more extensive populations (*e.g.* neonates, children, adults, specific disease conditions, and ethnicity) in comparison with non-company owned software tools. Automated population model selection based on the patient’s input data is activated in DoseMeRx, ID-ODS, InsightRX Nova, and PrecisePK. Published models have been selected in standardized ways before implementing in the software tools except for Tucuxi. However, the model selection procedures differ between software tools (*e.g.* published model from the peer-review journal, demographics of study participants, graphical or numerical goodness of fit, and simulation diagnostics). Models with inter-occasion variability are incorporated into five software tools (InsightRX Nova, MwPharm++, NextDose, PrecisePK, and Tucuxi). Users are allowed to define models and model parameter values in four software tools (MwPharm++, PrecisePK, Tucuxi, and TDMx). Model refinements with data collected from the intended clinical use are also possible for all the company software tools, AutoKinetics, and ID-ODS.

All of the software tools are capable of proposing *a priori* and *a posteriori* dosing regimens and also of handling non-steady state and irregular situations. Therapeutic target values are prespecified in eight software tools, except BestDose and PrecisePK. Users can also define their own target values in all software tools excluding AutoKinetics. The probability of target attainment is calculated and reported in five software tools (BestDose, ID-ODS, InsightRX Nova, MwPharm++, and TDMx). However, the user cannot define the desired probability of target attainment in any software. Concentration simulation with a specified dosing regimen is possible in all software tools except AutoKinetics. Also, the optimal sampling time point module is available in three software tools (BestDose, MwPharm++, and TDMx).

### Quality and Validation

The evaluated software tools fulfilled the quality and validation criteria for 76% (49–92%). DoseMeRx (92%), MwPharm++ (91%), and InsightRX Nova (90%) were three software tools that fulfilled most of the considered criteria.

A multidisciplinary team has been involved in all software developments (healthcare professionals, academic researchers, and computer experts). Only InsightRX Nova has involved the pharmaceutical industry in its development team. Seven software tools, except for BestDose, MwPharm++, and PrecisePK, verified their optimization algorithm against well-established mathematical software tools: NONMEM (AutoKinetics, InsightRx Nova, TDMx, and Tucuxi), R (AutoKinetics), Matlab (ID-ODS), and GNU Scientific Library (DoseMeRx). All EHR-integrated software tools validate the data exchange between software tools and the EHR, except for Tucuxi. All software tools are validated in the clinical setting in which they are intended to be deployed, except for NextDose and Tucuxi. Moreover, the software performance is continuously monitored once deployed in the clinical setting for all the company software tools, AutoKinetics, and NextDose.

In addition to software validation, model qualification has been performed by most evaluated software tools excluding Tucuxi. The selected models have been qualified for “fit for purpose” (*i.e.*, *a priori* and *a posteriori* predictive performance) by using various diagnostic tools such as visual predictive check and forecasting imprecision and bias. The model qualifications have been done by using not only external datasets but also historical data drawn from records of the clinical setting in which the software is intended to be used. A scientific publication of the implemented models is referred to in all software tools except for BestDose. To date, two software tools are CE-marked and registered as a medical device in Europe (*i.e.*, DoseMeRx and MwPharm++). In addition, DoseMeRx registered as a medical device in Australia.

### Output Generation

The evaluated software tools fulfilled the output generation criteria for 82% (39–95%). Tucuxi (95%), PrecisePK (90%), InsightRX Nova (88%), DoseMeRx (88%), and NextDose (88%) were four software tools that fulfilled most considered criteria. Differences between software tools are mostly explained by formats of recommended dosing regimen and report and capability of report generation.

A recommended dosing regimen is the primary output of MIPD software tools. PrecisePK, NextDose, and InsightRX Nova scored highest regarding a straightforward and easy to understand recommended dosing regimen. In contrast with other software tools, InsightRX Nova only outputs a dosing regimen table instead of a recommended dosing regimen. Their users can select the best dosing regimen based on the output table. Users can also customize the recommended dosing regimen (*e.g.* dosing interval) from most of the software tools except AutoKinetics and ID-ODS. In addition to the recommended dosing regimen, all software tools report individual PK parameters and generate a PK plot.

Seven software tools can generate reports except for AutoKinetics, BestDose, and the benchmarked versions of TDMx. All the reports are customizable and can be converted to PDF format. Reports from InsightRX Nova, PrecisePK, and DoseMeRx scored highest regarding readability.

### Privacy and Data Security

The evaluated software tools fulfilled the privacy and data security criteria for 88% (25–100%). The software tools provided by software companies and AutoKinetics fulfilled all the considered criteria (100%). Software tools differ mostly in terms of compliance to privacy policies and data security. All software tools except BestDose and ID-ODS informed that their software tools comply with European Union General Data Protection Regulation (EU GDPR) or equivalents. DoseMeRx and MwPharm++, two certified software-based medical devices, have also clarified terms about data storage and management in their privacy policy to their users. Three of six software tools (DoseMeRx, InsightRX Nova, and PrecisePK) that are capable of data collection for model refinement comply with the Health Insurance Portability and Accountability Act (HIPAA) legislation. For the other three software tools (AutoKinetics, ID-ODS, and MwPharm++), either data anonymization or informed consent have been used in the data collection. For software tools with data storage capability, the databases are either encrypted or password-protected excluding the databases of BestDose. Also, multiple user accommodations with a personal login and secured password are possible in all software tools except the benchmarked versions of TDMx and Tucuxi.

### Cost

Four of the six non-company owned providers offer their software tool free of charge (ID-ODS, NextDose, TDMx, and Tucuxi). The other two non-company owned providers charge their users for maintenance and support contracts (AutoKinetics) and for software development and software hosting (BestDose). For company-provided software tools, their cost plans are flexible and customizable. Maintenance and support costs are covered in their license fees. Moreover, costs of all the software tools can vary depending on organization (*e.g.* based on the number of users, type [*i.e.*, academic, enterprise]) and desired functionalities (*e.g.* integration, cloud storage). Five software tools have performed a cost-effectiveness analysis of the software-based treatment in comparison with standard treatment (*i.e.*, AutoKinetics [part of the current ongoing trial (Roggeveen et al., 2019)], BestDose [(Neely et al., 2018)], DoseMeRx [as white papers], InsightRX Nova [as a white paper], and MwPharm++ [trial ongoing]).

## Discussion

This study is the first to comparatively evaluate the performances of MIPD software tools that are currently available worldwide since the benchmarking study by Fuchs et al. in 2013, based on both selection and evaluation criteria. During the past 7 years, we found that notable efforts have been put into the development of user-friendly, high-quality and highly-secured MIPD software tools. Nevertheless, the 10 evaluated software tools were widely different in terms of in-built software features, user interface design, number of drug modules and populations, user support, quality control, and cost. Furthermore, there is still a demand for EHR integration, standardization of software and model validation strategies, and prospective evidence for the software tools’ clinical and cost benefits.

There were substantial differences between the MIPD software tools evaluated in our study in comparison to those evaluated in two previous landmark studies in terms of (i) included software tools, (ii) type of software application, and (iii) improvement in user-friendliness and data storage capability. In 1993, Buffington et al. published a review on 13 “clinical PK software programs” that were commercially available in the United States (Buffington et al., 1993). They concluded that the reviewed software programs can assist in the analysis of plasma drug concentration data for medications that warrant therapeutic drug monitoring. Twenty years later, Fuchs et al. published a benchmarking study of 12 “therapeutic drug monitoring software tools” (Fuchs et al., 2013). Only four included software tools were from previous studies by Buffington et al. They concluded that a simple, flexible, and user-friendly MIPD software tool with capabilities of data storage and EHR integration is still in demand. All of the software tools reviewed by the two previous studies were desktop software, while eight software tools included in our study are web-based software. Web-based software can be run from any web browsers with an internet connection regardless of the operating system, instead of requiring local installation. Web-based software also allows users to always access the most recent version of the software. We observed an evolution towards intuitive, easy to use, customizable software tools, and providers offering extensive user support and training. These findings are in agreement with a recently published study evaluating the user-friendliness of three software tools (Kumar et al., 2019). Moreover, eight evaluated software tools are capable to store data with data security management.

The capability to integrate into EHRs facilitates MIPD software tool utilization (Neely, 2017; Vinks et al., 2019). The integrated software tool can then automatically retrieve all required data available in the hospital’s health records and send back the output. There is a significant increase in the number of software tools with EHR integration capability from only one out of 12 software tools in Fuchs et al. study (MwPharm) to six out of 10 software tools in our study. Moreover, all six EHR-integrated software tools comply with privacy regulations (*i.e.* EU GDPR or equivalent and HIPAA). However, differences in EHR and clinical workflow remain challenges for wide integration of MIPD software tools in routine clinical practice.

Most of the evaluated software tools’ providers pay attention to not only the quality of the software tool itself, but also to the quality of population models implemented in these tools. It is important to implement the most appropriate model for a specific patient/population that can predict a recommended dosing regimen precisely and with the lowest risk of bias. The models can be selected from either literature, be newly developed using data obtained from the intended population (Germovsek et al., 2016), or be a meta-model in case of well-studied drugs with a large number of published models (Knøsgaard et al., 2016; Claisse et al., 2019). The selected models should qualify for “fit for purpose” predictive performances (*i.e.*, *a priori* prediction and *a posteriori* prediction). Model qualifications for MIPD have been done by using an external dataset (Broeker et al., 2019), multiple external datasets (Guo et al., 2019), and case-specific dataset (Dhaese et al., 2019). However, specific model diagnostic tools for model qualification, that allow standardized evaluation, are still lacking (Keizer et al., 2018). The qualified model might be undermined by user-defined model features that are allowed in some of the evaluated MIPD software tools. Therefore, such features should be restricted to an experienced user. Moreover, the quality of data collected from the intended clinical use for model refinement should be taken into consideration.

`The quality system regulations for MIPD software tools in Europe and Australia differ from those in the United States. The European Commission (EC) and the Australian Register of Therapeutic Goods (ARTG) define software that provides information to be used in making decisions for treatment as a “medical device” (European Commission, 2017; Australian Register of Therapeutic Goods, 2019). Conversely, the United States Food and Drug Administration (FDA) classifies clinical decision support software regarding the software’s recommendation (U.S. Food and Drug Administration, 2019). Software that provides consistent recommendation with FDA-required labeling is considered as a “non-device clinical decision support software,” while there is still no regulation for software that recommends an off-label dosing regimen. Regarding user training requirement, EU medical device regulation requires both initial and ongoing training for software user (European Commission, 2017). To date, the only Bayesian software that has been registered in the United States is myPKFiT (Takeda Pharmaceutical Company Limited, Lexington, MA) for the precision dosing of factor VIII in the management of hemophilia A (Álvarez-Román et al., 2017). The myPKFiT software was co-developed by the pharmaceutical industry during drug development so that its suggested dose is consistent with the prescribing information. Moreover, it is a milestone software tool that has been widely adopted into routine clinical practice as a companion tool for drug prescribing (Polasek et al., 2019b).

Until today, MIPD software tools have not been widely integrated into routine clinical practice. There are various factors that have withheld the software tools from wider integration (Darwich et al., 2017; Polasek et al., 2019c; Wright et al., 2019). Firstly, evidence for its clinical and economic benefit generated from prospective randomized controlled trials is still lacking. To date, clinical trials to prospectively access clinical and cost saving impacts of the evaluated MIPD software tools have not been widely conducted [*e.g.* a desktop version of BestDose (Neely et al., 2018), the benchmarked version of TDMx (Olbrisch et al., 2019), and the ongoing trial of AutoKinetics (Roggeveen et al., 2019)]. However, both finished studies reported superior clinical benefits from utilizing the MIPD software tools. Secondly, the actual implementation of MIPD into clinical workflow is likely to be more complex (*e.g.* additional clinical visit for blood sampling, availability of rapid sample measurement, and flexibility of available drug dose) (Dreesen et al., 2017; Strik et al., 2018; Polasek et al., 2019b). To facilitate a wider integration of MIPD software tools into clinical practice, a group of patients, drug characteristics, and disease that are highly impacted by MIPD should be clearly defined so that resource allocation and the evidence of clinical utility grow more rapidly (Polasek et al., 2019d). Moreover, interdisciplinary collaborations between software providers, software purchasers (e.g. hospital executives), clinical end-users (*e.g.*, clinician, clinical pharmacist, and pharmacometrician), and regulators require to fulfill all sectors’ need of the MIPD software tool in practice.

In addition to the MIPD module, DoseMeRx and InsightRX Nova also offer broader functions to their users. DoseMeRx offers DoseMe Crunch as a big data mining tool for data analyzing, while InsightRX Nova offers additional innovative modules in its platform framework for continuous learning such as specialized analytic dashboards and human-assisted artificial intelligence. Moreover, recently, InsightRX Nova has partnered up with BestDose to incorporate BestDose’s non-parametric optimization algorithms and its models into the InsightRX Nova platform (InsightRX, 2019).

The focus of our study was not to recommend the best software tools, but rather to provide information about the features of currently available MIPD software tools. Although ranking software tools based on their benchmarking scores is an objective evaluation criterion and represents a sensible way of evaluating the “overall performance” of a software tool, this approach has several limitations. First, a better quantitative performance (fulfilling more benchmarking criteria) does not necessarily imply a better qualitative performance. For example, the more drug modules are available, the higher the benchmarking score assigned to the software tool. However, a potential end-user may only be interested in one or a few specific drug modules. Also, the software providers that perform model validation before integration into the software tool receive a higher benchmarking score. However, model validation procedures are not standardized and may differ in quality. Second, the specific needs of a certain end-user are not necessarily fulfilled by the software tool with the best overall performance. An MIPD software tool that fulfilled more of the considered criteria may have been assigned a higher benchmarking score, but this does not necessarily mean that the software tool is the “best” for each and every end-user. Therefore, the overall performance scores may not be the best guide for selecting an MIPD software tool that needs to fit a specific clinical setting and end-user’s needs. Instead of a software tool ranking, it was our ambition to give an overview of all features, providing tailored guidance to the reader when selecting a software tool. This study has some limitations. First, we only evaluated one version and type of software application of each software tool. It may be that functions are not available in other versions and vice versa. Second, the AutoKinetics software was evaluated based on a one-hour web meeting with the providers because the software is only available as the EHR-integrated version. Third, some of the evaluation criteria could not be tested by the researchers, for example, the capability of EHR integration, model qualification, and model selection procedures before implementing models into the software, and verification of software optimization algorithm. For those criteria we relied on the available information on their websites, the information in previous literature, filled-out answers by the software providers, a one-hour online introduction with the software providers (DoseMeRx, PrecisePK, InsightRX Nova, and AutoKinetics), and email responses from the providers. Fourth, as opposed to Fuchs et al., we did not test software tools with real clinical precision dosing cases. Nevertheless, most of the currently proposed minimum quality standard considerations for pharmacokinetic calculators for drug dose individualization were included in our evaluation grid (Janković, 2019). This was evidenced by the fact that the experts did not suggest any additional evaluation criteria in this study. Moreover, in comparison with the previous benchmarking study by Fuchs et al., this study included a higher number of experts (22 as opposed to 15 in the study by Fuchs et al.). We consulted pharmacometricians instead of computer engineers in the field of precision dosing. Moreover, the software tool was evaluated by four researchers (two pharmacists and two pharmacometricians) instead of one pharmacist in the study by Fuchs et al.

To conclude, based on our findings, we believed that future work should focus on the standardization of software validation, model selection, and model validation in MIPD software tool development. While today these strategies widely differ between software tools, harmonization of these processes will allow a better comparison between different MIPD initiatives and will hopefully unambiguously demonstrate its clinical value. Joint efforts from software providers, academic researchers, and regulators are therefore required to stimulate this standardization, and facilitate a wider integration of MIPD software tools into clinical practice.

## Conclusion

To conclude, this study provides important insight into the comparative performance of currently available MIPD software tools and their requirements. All software tools in our study performed well in all the evaluated categories. With these overall positive results, it is anticipated that wider implementation of these software tools will increase in routine clinical practice. However, the establishment of an MIPD-centered healthcare workflow requires not only a state-of-the-art software tool but also other crucial components such as point-of-care assays and flexibility of drug dose and label.

## Data Availability Statement

The datasets generated for this study are available on request to the corresponding author.

## Ethics Statement

Not required to obtain patient consent and ethics approval according to institutional guidelines and national legislation.

## Author Contributions

WK, RD, MG, IS, ED: designed the research. WK, RD, MG, ED: assembled the data and performed the research. WK, ED: analyzed the data and wrote the manuscript. All authors: critical revision of the manuscript and approved the final version of the manuscript.

## Funding

This publication was made possible through funding support of the KU Leuven Fund for Fair Open Access. ED is a postdoctoral research fellow of the Research Foundation–Flanders (FWO), Belgium, Grant number: 12X9420N.

## Conflict of Interest

IS received financial support from Pfizer, Gilead, Merck Sharp & Dohme and Cidara. MF received financial support for research from Amgen, Biogen, Janssen Pharmaceutica, Pfizer, Takeda, lecture fees from Abbvie, Amgen, Biogen, Boehringer-Ingelheim, Falk, Ferring, Janssen Pharmaceutica, Lamepro, Merck Sharp & Dohme, Mylan, Pfizer, Takeda, and consultancy fees from Abbvie, Boehringer-Ingelheim, Janssen Pharmaceutica, Merck Sharp & Dohme, Pfizer, Sandoz, Takeda. ED has served as a speaker for Janssen Pharmaceutica and has served as an adviser for argenx (all honoraria/fees paid to the University). RD has received travel support from Pfizer, Inc. and Gilead Sciences.

The remaining authors declare that the research was conducted in the absence of any commercial or financial relationships that could be construed as a potential conflict of interest.
