# Supplementary material for: Software Tools for Model-Informed Precision Dosing: How Well Do They Satisfy the Needs?
Source: Front Pharmacol. 2020 May 7;11:620. doi: 10.3389/fphar.2020.00620 (PMC7224248; doi:10.3389/fphar.2020.00620)
Supplement: Supplementary file 1 [file DataSheet_1.docx]

Supplementary Material

# Supplementary Data

## Supplementary Data 1: An expert survey

The survey consisted of two parts. The first part of the survey obtained general information of the respondent. The second part consisted of questions designed to obtain participants’ perceptions regarding the importance of each evaluation criterion in MIPD software tool evaluation. The evaluation criteria were categorized into eight aspects as follows: user-friendliness and utilization, user support, computational aspect, population models, quality and validation, output and report generation, privacy and data security, and cost. The asterisks (*) were used for required fields.

**Consent Form**

Thank you for participating in this benchmarking study of model-informed precision dosing (MIPD) software tools.

MIPD software tools can be used to support decision-making on dose optimization for individual patients. The purpose of this study is to benchmark the currently available MIPD software tools.

The goal of this questionnaire is to document which criteria are important to consider when evaluating MIPD software tools for use in clinical practice. We would like to ask you to score different benchmarking criteria from 0 to 10 (with 0 being “I do not agree at all that this criterion is important” and 10 being “I agree completely that this criterion is important”). There is also an option saying “undecided”, e.g. when you think that your level of knowledge is not sufficient to evaluate this criterion. The questionnaire consists of two parts:

- Part 1 General information of the respondent
- Part 2 Features of the software tool in eight topics as follows:
  1. User-friendliness and utilization
  2. User support
  3. Computational aspect
  4. Population models
  5. Quality and validation
  6. Output and report generation
  7. Privacy and data security
  8. Cost

At the end of every topic, you have the possibility to enter some additional criteria or remarks that we should include in benchmarking of the MIPD software tools. It should take around 30 minutes to fill in this questionnaire.

Your answers and the results of this study will be used for research purposes only and will also be submitted for publication. Your answers will be pseudonymized and kept confidential. It will not be possible to identify who provided which answers in any publication.

If you have any questions about this survey, please contact: [wannee.kantasiripitak@kuleuven.be](mailto:wannee.kantasiripitak@kuleuven.be)

Do you consent that the answers provided in this survey will be used in the above- mentioned research study with the purpose of publication?*

🞅 Yes, I consent.

🞅 No, I do not consent.

Do you want your name to be mentioned in the acknowledgement section of the publication?*

🞅 Yes

🞅 No

Please fill in your name:*

…………………

**Part 1 General information of the respondent**

1.1 I am working as a:*

🞅 Pharmacometrician

🞅 Pharmacist

🞅 Clinician

Please specify your field of expertise.

…………………

- 1. On average, how often do you participate in clinical decision making based on therapeutic

drug monitoring?*

Never Rarely Monthly Weekly Daily

Frequency: 🞅 🞅 🞅 🞅 🞅

Please specify the concerned drug(s) included in your therapeutic drug monitoring programs.

…………………

**Part 2 Features of the software tool**

**2.1 User friendliness and utilization**

2.1.1 The software should support various operating systems (e.g. Mac, Windows, Linux).

do not agree at all 0 1 2 3 4 5 6 7 8 9 10 agree completely

🞅 🞅 🞅 🞅 🞅 🞅 🞅 🞅 🞅 🞅 🞅

□ undecided

2.1.2 A mobile platform application of the software should be available.

do not agree at all 0 1 2 3 4 5 6 7 8 9 10 agree completely

🞅 🞅 🞅 🞅 🞅 🞅 🞅 🞅 🞅 🞅 🞅

□ undecided

2.1.3 The software user should be able to download the software online.

do not agree at all 0 1 2 3 4 5 6 7 8 9 10 agree completely

🞅 🞅 🞅 🞅 🞅 🞅 🞅 🞅 🞅 🞅 🞅

□ undecided

2.1.4 The software should be easy to install.

do not agree at all 0 1 2 3 4 5 6 7 8 9 10 agree completely

🞅 🞅 🞅 🞅 🞅 🞅 🞅 🞅 🞅 🞅 🞅

□ undecided

2.1.5 A network installation should be provided by the software provider.

do not agree at all 0 1 2 3 4 5 6 7 8 9 10 agree completely

🞅 🞅 🞅 🞅 🞅 🞅 🞅 🞅 🞅 🞅 🞅

□ undecided

2.1.6 The software should be available in multiple languages.

do not agree at all 0 1 2 3 4 5 6 7 8 9 10 agree completely

🞅 🞅 🞅 🞅 🞅 🞅 🞅 🞅 🞅 🞅 🞅

□ undecided

2.1.7 The software user should be able to request an additional user interface language that is not yet supported by the software.

do not agree at all 0 1 2 3 4 5 6 7 8 9 10 agree completely

🞅 🞅 🞅 🞅 🞅 🞅 🞅 🞅 🞅 🞅 🞅

□ undecided

2.1.8 The software should be available as standalone software.

do not agree at all 0 1 2 3 4 5 6 7 8 9 10 agree completely

🞅 🞅 🞅 🞅 🞅 🞅 🞅 🞅 🞅 🞅 🞅

□ undecided

2.1.9 The software should be able to interface with hospital information systems.

do not agree at all 0 1 2 3 4 5 6 7 8 9 10 agree completely

🞅 🞅 🞅 🞅 🞅 🞅 🞅 🞅 🞅 🞅 🞅

□ undecided

2.1.10 Manual entry of data in the software should be possible.

do not agree at all 0 1 2 3 4 5 6 7 8 9 10 agree completely

🞅 🞅 🞅 🞅 🞅 🞅 🞅 🞅 🞅 🞅 🞅

□ undecided

2.1.11 Data import from a remote central database (e.g. hospital information systems) should be possible.

do not agree at all 0 1 2 3 4 5 6 7 8 9 10 agree completely

🞅 🞅 🞅 🞅 🞅 🞅 🞅 🞅 🞅 🞅 🞅

□ undecided

2.1.12 Data output and storage in a remote central database (e.g. hospital information systems) should be possible.

do not agree at all 0 1 2 3 4 5 6 7 8 9 10 agree completely

🞅 🞅 🞅 🞅 🞅 🞅 🞅 🞅 🞅 🞅 🞅

□ undecided

2.1.13 It should be possible to store input data entries and generated outputs in the software.

do not agree at all 0 1 2 3 4 5 6 7 8 9 10 agree completely

🞅 🞅 🞅 🞅 🞅 🞅 🞅 🞅 🞅 🞅 🞅

□ undecided

2.1.14 A database search by file name, patient name, patient identification, drug name, and/or date should be possible.

do not agree at all 0 1 2 3 4 5 6 7 8 9 10 agree completely

🞅 🞅 🞅 🞅 🞅 🞅 🞅 🞅 🞅 🞅 🞅

□ undecided

2.1.15 Each individual user account should be able to create their own patient database.

do not agree at all 0 1 2 3 4 5 6 7 8 9 10 agree completely

🞅 🞅 🞅 🞅 🞅 🞅 🞅 🞅 🞅 🞅 🞅

□ undecided

2.1.16 Add-on calculators (e.g. unit conversion, statistic calculation) should be available in the software.

do not agree at all 0 1 2 3 4 5 6 7 8 9 10 agree completely

🞅 🞅 🞅 🞅 🞅 🞅 🞅 🞅 🞅 🞅 🞅

□ undecided

2.1.17 The software interface should be visually appealing.

do not agree at all 0 1 2 3 4 5 6 7 8 9 10 agree completely

🞅 🞅 🞅 🞅 🞅 🞅 🞅 🞅 🞅 🞅 🞅

□ undecided

2.1.18 The software interface should be customizable according to the individual user’s needs.

do not agree at all 0 1 2 3 4 5 6 7 8 9 10 agree completely

🞅 🞅 🞅 🞅 🞅 🞅 🞅 🞅 🞅 🞅 🞅

□ undecided

2.1.19 There should be a wizard step-by-step interface for inexperienced software users.

do not agree at all 0 1 2 3 4 5 6 7 8 9 10 agree completely

🞅 🞅 🞅 🞅 🞅 🞅 🞅 🞅 🞅 🞅 🞅

□ undecided

2.1.20 It should be easy to enter the required patient data.

do not agree at all 0 1 2 3 4 5 6 7 8 9 10 agree completely

🞅 🞅 🞅 🞅 🞅 🞅 🞅 🞅 🞅 🞅 🞅

□ undecided

2.1.21 Prior training should not be required to be able to work with the software.

do not agree at all 0 1 2 3 4 5 6 7 8 9 10 agree completely

🞅 🞅 🞅 🞅 🞅 🞅 🞅 🞅 🞅 🞅 🞅

□ undecided

Additional criteria regarding user friendliness and utilization:

…………………

**2.2 User support**

2.2.1 There should be a clinical manual available to the software user.

do not agree at all 0 1 2 3 4 5 6 7 8 9 10 agree completely

🞅 🞅 🞅 🞅 🞅 🞅 🞅 🞅 🞅 🞅 🞅

□ undecided

2.2.2 There should be a technical manual available to the software user.

do not agree at all 0 1 2 3 4 5 6 7 8 9 10 agree completely

🞅 🞅 🞅 🞅 🞅 🞅 🞅 🞅 🞅 🞅 🞅

□ undecided

2.2.3 There should be a help desk or web support service, providing timely and useful support.

do not agree at all 0 1 2 3 4 5 6 7 8 9 10 agree completely

🞅 🞅 🞅 🞅 🞅 🞅 🞅 🞅 🞅 🞅 🞅

□ undecided

2.2.4 There should be a large and active community of software users (e.g. an online forum) that brings software users in contact with each other.

do not agree at all 0 1 2 3 4 5 6 7 8 9 10 agree completely

🞅 🞅 🞅 🞅 🞅 🞅 🞅 🞅 🞅 🞅 🞅

□ undecided

2.2.5 There should be training available for software users (e.g. on-site training, online training).

do not agree at all 0 1 2 3 4 5 6 7 8 9 10 agree completely

🞅 🞅 🞅 🞅 🞅 🞅 🞅 🞅 🞅 🞅 🞅

□ undecided

2.2.6 The software user should be able to update the software easily.

do not agree at all 0 1 2 3 4 5 6 7 8 9 10 agree completely

🞅 🞅 🞅 🞅 🞅 🞅 🞅 🞅 🞅 🞅 🞅

□ undecided

Additional criteria regarding user support:

…………………

**2.3 Computational aspect**

2.3.1 The previous versions of the software should stay accessible.

do not agree at all 0 1 2 3 4 5 6 7 8 9 10 agree completely

🞅 🞅 🞅 🞅 🞅 🞅 🞅 🞅 🞅 🞅 🞅

□ undecided

2.3.2 A list of changes and bug fixes between versions should be documented.

do not agree at all 0 1 2 3 4 5 6 7 8 9 10 agree completely

🞅 🞅 🞅 🞅 🞅 🞅 🞅 🞅 🞅 🞅 🞅

□ undecided

2.3.3 The source code should be accessible by the software user.

do not agree at all 0 1 2 3 4 5 6 7 8 9 10 agree completely

🞅 🞅 🞅 🞅 🞅 🞅 🞅 🞅 🞅 🞅 🞅

□ undecided

2.3.4 A runtime framework support should not be necessary for executing the software.

do not agree at all 0 1 2 3 4 5 6 7 8 9 10 agree completely

🞅 🞅 🞅 🞅 🞅 🞅 🞅 🞅 🞅 🞅 🞅

□ undecided

2.3.5 The minimum memory requirement to run the software should be taken into account.

do not agree at all 0 1 2 3 4 5 6 7 8 9 10 agree completely

🞅 🞅 🞅 🞅 🞅 🞅 🞅 🞅 🞅 🞅 🞅

□ undecided

2.3.6 There should be verbose bug outputs (i.e. messages about errors and warning conditions) from the software to the software user.

do not agree at all 0 1 2 3 4 5 6 7 8 9 10 agree completely

🞅 🞅 🞅 🞅 🞅 🞅 🞅 🞅 🞅 🞅 🞅

□ undecided

2.3.7 The graphic user interface (GUI) tools should be available.

do not agree at all 0 1 2 3 4 5 6 7 8 9 10 agree completely

🞅 🞅 🞅 🞅 🞅 🞅 🞅 🞅 🞅 🞅 🞅

□ undecided

2.3.8 There should be high capacity for structured data import.

do not agree at all 0 1 2 3 4 5 6 7 8 9 10 agree completely

🞅 🞅 🞅 🞅 🞅 🞅 🞅 🞅 🞅 🞅 🞅

□ undecided

2.3.9 There should be high capacity for structured data export.

do not agree at all 0 1 2 3 4 5 6 7 8 9 10 agree completely

🞅 🞅 🞅 🞅 🞅 🞅 🞅 🞅 🞅 🞅 🞅

□ undecided

Additional criteria regarding computational aspect:

…………………

**2.4 Population models**

2.4.1 The number of drugs and drug classes included in the software is relevant.

do not agree at all 0 1 2 3 4 5 6 7 8 9 10 agree completely

🞅 🞅 🞅 🞅 🞅 🞅 🞅 🞅 🞅 🞅 🞅

□ undecided

2.4.2 The populations for which the models are developed are relevant.

do not agree at all 0 1 2 3 4 5 6 7 8 9 10 agree completely

🞅 🞅 🞅 🞅 🞅 🞅 🞅 🞅 🞅 🞅 🞅

□ undecided

2.4.3 Suitable diagnostic tools and/or methods should be used in model selection prior to implementing a model in the software.

do not agree at all 0 1 2 3 4 5 6 7 8 9 10 agree completely

🞅 🞅 🞅 🞅 🞅 🞅 🞅 🞅 🞅 🞅 🞅

□ undecided

2.4.4 The software should be able to select the model automatically based on patient data that are imported.

do not agree at all 0 1 2 3 4 5 6 7 8 9 10 agree completely

🞅 🞅 🞅 🞅 🞅 🞅 🞅 🞅 🞅 🞅 🞅

□ undecided

2.4.5 The information on model selection should be available for the software user.

do not agree at all 0 1 2 3 4 5 6 7 8 9 10 agree completely

🞅 🞅 🞅 🞅 🞅 🞅 🞅 🞅 🞅 🞅 🞅

□ undecided

2.4.6 The drug-specific references of the models used in the software, should be available.

do not agree at all 0 1 2 3 4 5 6 7 8 9 10 agree completely

🞅 🞅 🞅 🞅 🞅 🞅 🞅 🞅 🞅 🞅 🞅

□ undecided

2.4.7 Models with inter-occasion variability should be incorporated into the software.

do not agree at all 0 1 2 3 4 5 6 7 8 9 10 agree completely

🞅 🞅 🞅 🞅 🞅 🞅 🞅 🞅 🞅 🞅 🞅

□ undecided

2.4.8 The software should be able to perform non-Bayesian calculations.

do not agree at all 0 1 2 3 4 5 6 7 8 9 10 agree completely

🞅 🞅 🞅 🞅 🞅 🞅 🞅 🞅 🞅 🞅 🞅

□ undecided

2.4.9 The software should be able to perform Bayesian analysis.

do not agree at all 0 1 2 3 4 5 6 7 8 9 10 agree completely

🞅 🞅 🞅 🞅 🞅 🞅 🞅 🞅 🞅 🞅 🞅

□ undecided

2.4.10 The software should be able to propose an a priori dosing regimen (i.e. dosing regimen proposal based on patients’ covariates).

do not agree at all 0 1 2 3 4 5 6 7 8 9 10 agree completely

🞅 🞅 🞅 🞅 🞅 🞅 🞅 🞅 🞅 🞅 🞅

□ undecided

2.4.11 The software should be able to propose an a priori loading dose (i.e. loading dose proposal based on patients’ covariates).

do not agree at all 0 1 2 3 4 5 6 7 8 9 10 agree completely

🞅 🞅 🞅 🞅 🞅 🞅 🞅 🞅 🞅 🞅 🞅

□ undecided

2.4.12 The software should be able to calculate and report the probability of target attainment.

do not agree at all 0 1 2 3 4 5 6 7 8 9 10 agree completely

🞅 🞅 🞅 🞅 🞅 🞅 🞅 🞅 🞅 🞅 🞅

□ undecided

2.4.13 The software user should be able to define the probability of target attainment.

do not agree at all 0 1 2 3 4 5 6 7 8 9 10 agree completely

🞅 🞅 🞅 🞅 🞅 🞅 🞅 🞅 🞅 🞅 🞅

□ undecided

2.4.14 The software should be able to propose an a posteriori dosing regimen (i.e. dosing regimen proposal based on drug measurement).

do not agree at all 0 1 2 3 4 5 6 7 8 9 10 agree completely

🞅 🞅 🞅 🞅 🞅 🞅 🞅 🞅 🞅 🞅 🞅

□ undecided

2.4.15 The software should be able to propose an a posteriori re-loading dose (i.e. re- loading dose proposal based on drug measurement) by taking into account an interruption in treatment.

do not agree at all 0 1 2 3 4 5 6 7 8 9 10 agree completely

🞅 🞅 🞅 🞅 🞅 🞅 🞅 🞅 🞅 🞅 🞅

□ undecided

2.4.16 The number of drug measurements that can be entered for an a posteriori dosing regimens proposal should be high or, ideally, unlimited.

do not agree at all 0 1 2 3 4 5 6 7 8 9 10 agree completely

🞅 🞅 🞅 🞅 🞅 🞅 🞅 🞅 🞅 🞅 🞅

□ undecided

2.4.17 The software should also consider drug measurements both below and above the limit of quantification.

do not agree at all 0 1 2 3 4 5 6 7 8 9 10 agree completely

🞅 🞅 🞅 🞅 🞅 🞅 🞅 🞅 🞅 🞅 🞅

□ undecided

2.4.18 The software should be able to use only the first dose and extrapolate this to steady state.

do not agree at all 0 1 2 3 4 5 6 7 8 9 10 agree completely

🞅 🞅 🞅 🞅 🞅 🞅 🞅 🞅 🞅 🞅 🞅

□ undecided

2.4.19 The software should be able to handle non-steady state conditions and irregular regimens.

do not agree at all 0 1 2 3 4 5 6 7 8 9 10 agree completely

🞅 🞅 🞅 🞅 🞅 🞅 🞅 🞅 🞅 🞅 🞅

□ undecided

2.4.20 The software user should be able to define a new model in the software.

do not agree at all 0 1 2 3 4 5 6 7 8 9 10 agree completely

🞅 🞅 🞅 🞅 🞅 🞅 🞅 🞅 🞅 🞅 🞅

□ undecided

2.4.21 The software user should be able to parameterize covariates used in the software.

do not agree at all 0 1 2 3 4 5 6 7 8 9 10 agree completely

🞅 🞅 🞅 🞅 🞅 🞅 🞅 🞅 🞅 🞅 🞅

□ undecided

2.4.22 The software user should be able to choose model parameter values (e.g. population parameters, user-defined parameters).

do not agree at all 0 1 2 3 4 5 6 7 8 9 10 agree completely

🞅 🞅 🞅 🞅 🞅 🞅 🞅 🞅 🞅 🞅 🞅

□ undecided

2.4.23 The software should specify default boundaries for therapeutic targets (e.g. trough, peak, peak-trough, AUC, MIC, time in range).

do not agree at all 0 1 2 3 4 5 6 7 8 9 10 agree completely

🞅 🞅 🞅 🞅 🞅 🞅 🞅 🞅 🞅 🞅 🞅

□ undecided

2.4.24 The software user should be able to define the boundaries for therapeutic targets (e.g. trough, peak, peak-trough, AUC, MIC, time in range).

do not agree at all 0 1 2 3 4 5 6 7 8 9 10 agree completely

🞅 🞅 🞅 🞅 🞅 🞅 🞅 🞅 🞅 🞅 🞅

□ undecided

2.4.25 The software user should be able to adjust or specify the dosage and obtain concentration simulations.

do not agree at all 0 1 2 3 4 5 6 7 8 9 10 agree completely

🞅 🞅 🞅 🞅 🞅 🞅 🞅 🞅 🞅 🞅 🞅

□ undecided

2.4.26 The software should include an optimal sampling time module.

do not agree at all 0 1 2 3 4 5 6 7 8 9 10 agree completely

🞅 🞅 🞅 🞅 🞅 🞅 🞅 🞅 🞅 🞅 🞅

□ undecided

2.4.27 The software user should be able to update the model structure or model parameters with data collected in the software to match the intended clinical use (i.e. model refinement).

do not agree at all 0 1 2 3 4 5 6 7 8 9 10 agree completely

🞅 🞅 🞅 🞅 🞅 🞅 🞅 🞅 🞅 🞅 🞅

□ undecided

Additional criteria regarding population models:

…………………

**2.5 Quality and validation**

2.5.1 Computer scientists should have been involved in software development.

do not agree at all 0 1 2 3 4 5 6 7 8 9 10 agree completely

🞅 🞅 🞅 🞅 🞅 🞅 🞅 🞅 🞅 🞅 🞅

□ undecided

2.5.2 Computer engineers should have been involved in software development.

do not agree at all 0 1 2 3 4 5 6 7 8 9 10 agree completely

🞅 🞅 🞅 🞅 🞅 🞅 🞅 🞅 🞅 🞅 🞅

□ undecided

2.5.3 Pharmacists should have been involved in software development.

do not agree at all 0 1 2 3 4 5 6 7 8 9 10 agree completely

🞅 🞅 🞅 🞅 🞅 🞅 🞅 🞅 🞅 🞅 🞅

□ undecided

2.5.4 Clinicians should have been involved in software development.

do not agree at all 0 1 2 3 4 5 6 7 8 9 10 agree completely

🞅 🞅 🞅 🞅 🞅 🞅 🞅 🞅 🞅 🞅 🞅

□ undecided

2.5.5 Pharmacometricians should have been involved in software development.

do not agree at all 0 1 2 3 4 5 6 7 8 9 10 agree completely

🞅 🞅 🞅 🞅 🞅 🞅 🞅 🞅 🞅 🞅 🞅

□ undecided

2.5.6 Academic institutions should have been involved in software development.

do not agree at all 0 1 2 3 4 5 6 7 8 9 10 agree completely

🞅 🞅 🞅 🞅 🞅 🞅 🞅 🞅 🞅 🞅 🞅

□ undecided

2.5.7 The pharmaceutical industry should have been involved in software development.

do not agree at all 0 1 2 3 4 5 6 7 8 9 10 agree completely

🞅 🞅 🞅 🞅 🞅 🞅 🞅 🞅 🞅 🞅 🞅

□ undecided

2.5.8 There should be a scientific publication describing the development of the software.

do not agree at all 0 1 2 3 4 5 6 7 8 9 10 agree completely

🞅 🞅 🞅 🞅 🞅 🞅 🞅 🞅 🞅 🞅 🞅

□ undecided

2.5.9 There should be a scientific publication describing the development of the models used in the software and the publication should be referenced to in the software

do not agree at all 0 1 2 3 4 5 6 7 8 9 10 agree completely

🞅 🞅 🞅 🞅 🞅 🞅 🞅 🞅 🞅 🞅 🞅

□ undecided

2.5.10 There should be a scientific publication describing clinical research using the software.

do not agree at all 0 1 2 3 4 5 6 7 8 9 10 agree completely

🞅 🞅 🞅 🞅 🞅 🞅 🞅 🞅 🞅 🞅 🞅

□ undecided

2.5.11 The model qualification should be performed for ‘fit for purpose’ prior to software implementation.

do not agree at all 0 1 2 3 4 5 6 7 8 9 10 agree completely

🞅 🞅 🞅 🞅 🞅 🞅 🞅 🞅 🞅 🞅 🞅

□ undecided

2.5.12 The model performance should be evaluated with historical data drawn from records of the clinical setting in which the software is intended to be used.

do not agree at all 0 1 2 3 4 5 6 7 8 9 10 agree completely

🞅 🞅 🞅 🞅 🞅 🞅 🞅 🞅 🞅 🞅 🞅

□ undecided

2.5.13 The selected model should be prospectively validated before implementation in the software.

do not agree at all 0 1 2 3 4 5 6 7 8 9 10 agree completely

🞅 🞅 🞅 🞅 🞅 🞅 🞅 🞅 🞅 🞅 🞅

□ undecided

2.5.14 The software optimization algorithm should be verified against a well-established mathematical software (e.g. NONMEM).

do not agree at all 0 1 2 3 4 5 6 7 8 9 10 agree completely

🞅 🞅 🞅 🞅 🞅 🞅 🞅 🞅 🞅 🞅 🞅

□ undecided

2.5.15 Data exchange should be validated in case the software interfaces with another external system.

do not agree at all 0 1 2 3 4 5 6 7 8 9 10 agree completely

🞅 🞅 🞅 🞅 🞅 🞅 🞅 🞅 🞅 🞅 🞅

□ undecided

2.5.16 There should be a clinical validation of the software.

do not agree at all 0 1 2 3 4 5 6 7 8 9 10 agree completely

🞅 🞅 🞅 🞅 🞅 🞅 🞅 🞅 🞅 🞅 🞅

□ undecided

2.5.17 The software should be approved as a software-based medical device with CE marking.

do not agree at all 0 1 2 3 4 5 6 7 8 9 10 agree completely

🞅 🞅 🞅 🞅 🞅 🞅 🞅 🞅 🞅 🞅 🞅

□ undecided

2.5.18 The software performance should be monitored continuously once deployed in the clinical setting.

do not agree at all 0 1 2 3 4 5 6 7 8 9 10 agree completely

🞅 🞅 🞅 🞅 🞅 🞅 🞅 🞅 🞅 🞅 🞅

□ undecided

Additional criteria regarding quality and validation:

…………………

**2.6 Output and report generation**

2.6.1 The software should suggest a list of best dose candidate(s).

do not agree at all 0 1 2 3 4 5 6 7 8 9 10 agree completely

🞅 🞅 🞅 🞅 🞅 🞅 🞅 🞅 🞅 🞅 🞅

□ undecided

2.6.2 The dosing recommendation from the software should be straightforward and easy to understand.

do not agree at all 0 1 2 3 4 5 6 7 8 9 10 agree completely

🞅 🞅 🞅 🞅 🞅 🞅 🞅 🞅 🞅 🞅 🞅

□ undecided

2.6.3 The dosing recommendation from the software should be customizable by the user.

do not agree at all 0 1 2 3 4 5 6 7 8 9 10 agree completely

🞅 🞅 🞅 🞅 🞅 🞅 🞅 🞅 🞅 🞅 🞅

□ undecided

2.6.4 Individual pharmacokinetic parameters should be reported.

do not agree at all 0 1 2 3 4 5 6 7 8 9 10 agree completely

🞅 🞅 🞅 🞅 🞅 🞅 🞅 🞅 🞅 🞅 🞅

□ undecided

2.6.5 A pharmacokinetic and/or pharmacodynamics plot should be generated.

do not agree at all 0 1 2 3 4 5 6 7 8 9 10 agree completely

🞅 🞅 🞅 🞅 🞅 🞅 🞅 🞅 🞅 🞅 🞅

□ undecided

2.6.6 It should be possible to add or remove the prediction intervals and/or percentiles on the plot.

do not agree at all 0 1 2 3 4 5 6 7 8 9 10 agree completely

🞅 🞅 🞅 🞅 🞅 🞅 🞅 🞅 🞅 🞅 🞅

□ undecided

2.6.7 The plot should be interactive and adjustable.

do not agree at all 0 1 2 3 4 5 6 7 8 9 10 agree completely

🞅 🞅 🞅 🞅 🞅 🞅 🞅 🞅 🞅 🞅 🞅

□ undecided

2.6.8 A report should be presented in an easily readable and customizable way and all essential information should be included.

do not agree at all 0 1 2 3 4 5 6 7 8 9 10 agree completely

🞅 🞅 🞅 🞅 🞅 🞅 🞅 🞅 🞅 🞅 🞅

□ undecided

2.6.9 The plot should be included in the report.

do not agree at all 0 1 2 3 4 5 6 7 8 9 10 agree completely

🞅 🞅 🞅 🞅 🞅 🞅 🞅 🞅 🞅 🞅 🞅

□ undecided

2.6.10 A text field (e.g. note box) in the report should be editable in the software directly.

do not agree at all 0 1 2 3 4 5 6 7 8 9 10 agree completely

🞅 🞅 🞅 🞅 🞅 🞅 🞅 🞅 🞅 🞅 🞅

□ undecided

2.6.11 The software user identity should be indicated in the report.

do not agree at all 0 1 2 3 4 5 6 7 8 9 10 agree completely

🞅 🞅 🞅 🞅 🞅 🞅 🞅 🞅 🞅 🞅 🞅

□ undecided

2.6.12 The software user should be able to convert the report to the desired format (e.g. PDF).

do not agree at all 0 1 2 3 4 5 6 7 8 9 10 agree completely

🞅 🞅 🞅 🞅 🞅 🞅 🞅 🞅 🞅 🞅 🞅

□ undecided

Additional criteria regarding output and report generation:

…………………

**2.7 Privacy and data security**

2.7.1 Software should comply with the European Union General Data Protection Regulation (EU GDPR) or equivalent.

do not agree at all 0 1 2 3 4 5 6 7 8 9 10 agree completely

🞅 🞅 🞅 🞅 🞅 🞅 🞅 🞅 🞅 🞅 🞅

□ undecided

2.7.2 Multiple users should be able to accommodate the software with a personal login and by a secured password.

do not agree at all 0 1 2 3 4 5 6 7 8 9 10 agree completely

🞅 🞅 🞅 🞅 🞅 🞅 🞅 🞅 🞅 🞅 🞅

□ undecided

2.7.3 The database should be encoded.

do not agree at all 0 1 2 3 4 5 6 7 8 9 10 agree completely

🞅 🞅 🞅 🞅 🞅 🞅 🞅 🞅 🞅 🞅 🞅

□ undecided

2.7.4 A data privacy method should be used in data collection.

do not agree at all 0 1 2 3 4 5 6 7 8 9 10 agree completely

🞅 🞅 🞅 🞅 🞅 🞅 🞅 🞅 🞅 🞅 🞅

□ undecided

Additional criteria regarding privacy and data security:

…………………

**2.8 Cost**

2.8.1 An individual license should be available at a reasonable cost.

do not agree at all 0 1 2 3 4 5 6 7 8 9 10 agree completely

🞅 🞅 🞅 🞅 🞅 🞅 🞅 🞅 🞅 🞅 🞅

□ undecided

2.8.2 The institution license should be available at a reduced cost.

do not agree at all 0 1 2 3 4 5 6 7 8 9 10 agree completely

🞅 🞅 🞅 🞅 🞅 🞅 🞅 🞅 🞅 🞅 🞅

□ undecided

2.8.3 An institution license should be available at a reasonable cost.

do not agree at all 0 1 2 3 4 5 6 7 8 9 10 agree completely

🞅 🞅 🞅 🞅 🞅 🞅 🞅 🞅 🞅 🞅 🞅

□ undecided

2.8.4 The software version with additional functions should be available at a reasonable cost.

do not agree at all 0 1 2 3 4 5 6 7 8 9 10 agree completely

🞅 🞅 🞅 🞅 🞅 🞅 🞅 🞅 🞅 🞅 🞅

□ undecided

2.8.5 The software maintenance and support contract should be available at a reasonable cost.

do not agree at all 0 1 2 3 4 5 6 7 8 9 10 agree completely

🞅 🞅 🞅 🞅 🞅 🞅 🞅 🞅 🞅 🞅 🞅

□ undecided

2.8.6 There should be a cost-effectiveness analysis of the software-based treatment in comparison with standard treatment.

do not agree at all 0 1 2 3 4 5 6 7 8 9 10 agree completely

🞅 🞅 🞅 🞅 🞅 🞅 🞅 🞅 🞅 🞅 🞅

□ undecided

Additional criteria regarding cost:

…………………

**Submission**

Thank you for your participation. Your answers will help us to evaluate the model-informed precision dosing software tools accurately.

## Supplementary Data 2: A software provider survey

The survey consisted of two parts. The first part of the survey obtained descriptive characteristics of the MIPD software tools. The second part consisted of questions designed to obtain features of the MIPD software tool in eight aspects (i.e., user-friendliness and utilization, user support, computational aspect, population models, quality and validation, output and report generation, privacy and data security, and cost). The asterisks (*) were used for required fields.

**Consent Form**

Thank you for accepting to participate in a benchmarking study of model-informed precision dosing software tools.

The purpose of this research study is to benchmark currently available model-informed precision dosing software tools. The procedure involves answering an online software developer questionnaire to provide information about the software tool. The questionnaire consists of two parts:

- Part 1 Descriptive characteristics of the software tool.
- Part 2 Features of the software tool in eight aspects as follows:
  1. User-friendliness and utilization
  2. User support
  3. Computational aspect
  4. Pharmacokinetics and model
  5. Quality and validation
  6. Output and report generation
  7. Privacy and data security
  8. Cost

The acquired information will be handled confidentially and used to evaluate the software tool. The acquired information and results of the study will be used for research purposes only and also submitted for publication. If you have any questions about the research study, please contact: [wannee.kantasiripitak@kuleuven.be](mailto:wannee.kantasiripitak@kuleuven.be)

Do you consent that the information that you provide in this survey will be used in the above-mentioned research study and possibly will be published?*

🞅 Yes, I consent.

🞅 No, I do not consent.

**Part 1 Descriptive characteristics of the software tool**

- 1. Software tool name:*

…………………

- 1. Company/institution:*

…………………

- 1. Location of company/institution:

…………………

- 1. Developer(s) of the provided software version:

…………………

- 1. Website:*

…………………

- 1. Computer language of source code:*

…………………

- 1. Release date of the first version:*

…………………

- 1. Is the software demo you provide to us the same version as the software on which you comment in this questionnaire?*

🞅 same version

🞅 Other …………………

**Part 2 Features of the software tool**

**2.1 User friendliness and utilization**

2.1.1 What are the purpose(s) of the software?*

🞅 Research purpose

🞅 Clinical purpose

🞅 Both research and clinical purpose

🞅 Other

Additional comment: …………………

2.1.2 Which platform is the software compatible with?*

🞅 Windows

🞅 Mac

🞅 Linux

🞅 Android

🞅 IOS

🞅 Other

Additional comment: …………………

2.1.3 Is there a mobile application of the software available?*

🞅 Yes

🞅 No

If yes, please provide the mobile application name.…………………

Additional comment: …………………

2.1.4 Can the software be downloaded online?*

🞅 Yes

🞅 No

If yes, please specify method to download the software.…………………

Additional comment: …………………

2.1.5 Is network installation provided by the company/institution?*

🞅 Yes

🞅 No

Additional comment: …………………

- - 1. How many languages does the software support?*

Please specify a list of language(s).* …………………

Additional comment: …………………

2.1.7Can the software user request an additional user interface language that is not yet supported by the software?*

🞅 Yes

🞅 No

Additional comment: …………………

- - 1. Can software be interfaced with hospital information systems?*

🞅 Yes

🞅 No

If yes, please provide interfacing details (i.e., integration engine)…………………

Additional comment: …………………

- - 1. Is data collection from a remote central database possible?*

🞅 Yes

🞅 No

Additional comment: …………………

- - 1. Is there a searchable database for recorded data?*

🞅 Yes

🞅 No

If yes, please specify how can the recorded data be searched by?

File name

□ Patient name

□ Patient identification

□ Drug name

□ Date

□ Other: …………………

Additional comment: …………………

- - 1. Is it possible for software users to create their own database?*

🞅 Yes

🞅 No

Additional comment: …………………

- - 1. Can input data and generated output data be stored in the software?*

🞅 Yes

🞅 No

If yes, please specify data that are stored.

□ Input data

□ Generated output data

□ Both input data and generated output data

□ Other: …………………

Additional comment: …………………

- - 1. Are there add-on calculator(s) available in the software (e.g. unit conversion)?*

🞅 Yes

🞅 No

If yes, please specify the list of add-on calculator(s).…………………

- - 1. Do the software users need training before being able to use the software?*

🞅 Yes

🞅 No

If yes, please select level of training.

□ Minimal training

□ Moderate training

□ Extensive training

□ Other: …………………

Additional comment: …………………

**2.2 User support**

2.2.1 Is there a clinical manual available to the software user?*

🞅 Yes

🞅 No

If yes, please provide detail on how the software users can access to the manual.

Additional comment: …………………

2.2.2 Is there a technical manual available to the software user?*

🞅 Yes

🞅 No

If yes, please provide detail on how the software users can access to the manual.

Additional comment: …………………

2.2.3 Is there a help desk or web support service?*

🞅 Yes

🞅 No

If yes, please specify type of service(s).

□ Web

□ Support

□ Help desk

□ Live chat

□ Other: …………………

Opening hours: …………………

Additional comment: …………………

2.2.4 Do you provide a discussion forum for the software users?*

🞅 Yes

🞅 No

If yes, please provide detail.…………………

Additional comment: …………………

2.2.5 Are there trainings available for the software user?*

🞅 Yes

🞅 No

If yes, please provide detail.…………………

Additional comment: …………………

2.2.6 How can the software user update the software?*

□ A software update is not possible.

□ The users is unable to check for software updates.

□ Via the company website.

□ Via an "update button" implemented in the software.

□ An update link will be sent via email to the software user.

□ Other: …………………

Additional comment: …………………

2.2.7 Does your company/institution provide user support in any other way than asked for in the above questions?

…………………

**2.3 Computational aspect**

2.3.1 Can the software user still access previous version(s) of the software?*

🞅 Yes

🞅 No

Additional comment: …………………

2.3.2 Is there an available list of changes and bug fixes between versions?*

🞅 Yes

🞅 No

Additional comment: …………………

2.3.3 Can the software user access the source code of the software?*

🞅 Yes

🞅 No

Additional comment: …………………

2.3.4 Is runtime framework support necessary for executing the software?*

🞅 Yes

🞅 No

If yes, please provide runtime framework support name.…………………

Additional comment: …………………

2.3.5 What is the minimum memory requirement to run the software?*

…………………

Additional comment: …………………

2.3.6 Are there verbose bug outputs available from the software to the software user?*

🞅 Yes

🞅 No

Additional comment: …………………

2.3.7 Can structured data be imported into the software?*

🞅 Yes

🞅 No

If yes, please specified the list of supported import file format(s).…………………

Additional comment: …………………

2.3.8 Can structured data be exported from the software?*

🞅 Yes

🞅 No

If yes, please specified the list of supported export file format(s)..…………………

Additional comment: …………………

**2.4 Population models**

2.4.1 How many drugs are currently available in the software?*

Please specify the list of drug(s).* …………………

Additional comment: …………………

2.4.2 Are standardized procedure(s) used in model selection prior to implementing a model in the software?*

🞅 Yes

🞅 No

If yes, please specify method(s)/diagnostic tool(s) used for model selection.…………………

Additional comment: …………………

2.4.3 Are the drug-specific references on the model parameter used available in the software?*

🞅 Yes

🞅 No

🞅 Other: …………………

Additional comment: …………………

2.4.4 At this point, are there any models with inter-occasion variability that have been incorporated in the software?*

🞅 Yes

🞅 No

If yes, please provide reference(s) of the model.…………………

Additional comment: …………………

2.4.5 Is the software capable of performing non-Bayesian calculations?*

🞅 Yes

🞅 No

Additional comment: …………………

2.4.6 Is the software capable of performing Bayesian analysis?*

🞅 Yes

🞅 No

Additional comment: …………………

2.4.7 Can the software be used to propose an a priori dosing regimen?*

🞅 Yes

🞅 No

Additional comment: …………………

2.4.8 Can the software be used to propose an a priori loading dose?*

🞅 Yes

🞅 No

Additional comment: …………………

2.4.9 Does the software calculate and report the probability of target attainment?*

🞅 Yes

🞅 No

Additional comment: …………………

2.4.10 Can the software be used to propose an a posteriori dosing regimen?*

🞅 Yes

🞅 No

If yes, please select the method used in dose optimization.

□ Linear regression analysis

□ Bayesian parametric approach

□ Bayesian non-parametric approach

□ Other: …………………

Additional comment: …………………

2.4.11 Can the software be used to propose an a posteriori re-loading dose?*

🞅 Yes

🞅 No

If yes, is an interruption in treatment also taken into account in the re-loading dose proposal?

🞅 Yes

🞅 No

Additional comment: …………………

2.4.12 How many drug measurements can be entered for a posteriori dosing regimen proposals?*

🞅 1 drug measurement

🞅 2 drug measurements

🞅 3 drug measurements

🞅 Unlimited number of drug measurements

🞅 Other: …………………

Additional comment: …………………

2.4.13 Dose the software consider drug measurements below the limit of quantification?*

🞅 Yes

🞅 No

Additional comment: …………………

2.4.14 Can the software extrapolate the first dose to steady state?*

🞅 Yes

🞅 No

Additional comment: …………………

2.4.15 Can the software handle non-steady state situations and/or irregular regimens?*

🞅 Non-steady state situations can be handled.

🞅 Irregular regimens can be handled.

🞅 Both non-steady state situations and irregular regimens can be handled.

Additional comment: …………………

2.4.16 Can the software user define a new model in the software?*

🞅 Yes

🞅 No

Additional comment: …………………

2.4.17 Can the software user parameterize covariates used in the model?*

🞅 Yes

🞅 No

Additional comment: …………………

2.4.18 Can the software user input parameter values used in the model?*

🞅 Yes

🞅 No

If yes, please specify the input method.

🞅 By changing the value in the appropriate field

🞅 By ticking "user-defined box" with visible default population parameter values

🞅 Other: …………………

Additional comment: …………………

2.4.19 Can the software user adjust or specify the dosage and obtain concentration simulation?*

🞅 Yes

🞅 No

Additional comment: …………………

2.4.20 Does the software include an optimal sampling time module?*

🞅 Yes

🞅 No

If yes, please specify method(s) used for the module.…………………

Additional comment: …………………

2.4.21 Can model refinement be performed in the software by using collected data after utilizing the software in practice?*

🞅 Yes

🞅 No

Additional comment: …………………

**2.5 Quality and validation**

2.5.1 Which experts have been involved in development of the provided software version?*

□ Computer scientist(s)

□ Computer engineer(s)

□ Pharmacist(s)

□ Clinician(s)

□ Academic institution

□ Pharmaceutical industry

□ Other: …………………

Additional comment: …………………

2.5.2 Is there a scientific publication describing the development of the software?*

🞅 Yes

🞅 No

If yes, please specify the publication.…………………

Additional comment: …………………

2.5.3 Are there scientific publications describing the development of the models used in the software?*

🞅 Yes

🞅 No

If yes, are the publications referred to in the software?

🞅 Yes

🞅 No

Additional comment: …………………

2.5.4 Has model qualification been performed prior to software implementation (e.g. a priori predictive performance, a posteriori predictive performance)?*

🞅 Yes

🞅 No

Additional comment: …………………

2.5.5 Has model performance been evaluated with historical data drawn from the clinical record of the clinical setting in which the software is going to be implemented?*

🞅 Yes

🞅 No

Additional comment: …………………

2.5.6 Has prospective validation of the selected model been performed prior to software implementation?*

🞅 Yes

🞅 No

Additional comment: …………………

2.5.7 Has the software optimization algorithm been verified against a well-established mathematical software?*

🞅 Yes

🞅 No

If yes, please specify the mathematical software.…………………

Additional comment: …………………

2.5.8 Is there a validation for data exchange when interfacing with another external system?*

🞅 Yes

🞅 No

Additional comment: …………………

2.5.9 Is there a clinical validation of the software in the clinical setting?*

🞅 Yes

🞅 No

Additional comment: …………………

2.5.10 Has the company submitted an application for approval of the software as a medical device?*

🞅 Yes

🞅 No

If yes, please specify status of approval.

🞅 Medical device certification received.

🞅 The application is ongoing.

🞅 Other: …………………

If no, is there any plan for submitting such an application.

🞅 Yes

🞅 No

Additional comment: …………………

2.5.11 Has the company/institution continuously monitored the software once deployed in the clinical setting?*

🞅 Yes

🞅 No

If yes, please briefly describe the software monitoring procedure.…………………

Additional comment: …………………

**2.6 Output an report generation**

2.6.1 Does the software suggest a list of best dose candidate(s) for dose optimization?*

🞅 Yes

🞅 No

Additional comment: …………………

2.6.2 Does the software calculate and report individual pharmacokinetic parameters?*

🞅 Yes

🞅 No

Additional comment: …………………

2.6.3 Can the software generate a pharmacokinetic plot?*

🞅 Yes

🞅 No

Additional comment: …………………

2.6.4 Can prediction intervals and/or percentiles be displayed on the pharmacokinetic plot?*

🞅 Yes

🞅 No

Additional comment: …………………

2.6.5 Can the software generate a report?*

🞅 Yes

🞅 No

If yes, is the report customizable?

🞅 Yes

🞅 No

🞅 Other: …………………

Additional comment: …………………

2.6.6 Which user identities are displayed on the report?*

□ No user identity displays in the report.

□ Login name

□ Name of Consultant

□ Institution logo

□ Other: …………………

Additional comment: …………………

2.6.7 Can the software user convert reports to the desired format (e.g. PDF)?*

🞅 Yes

🞅 No

Additional comment: …………………

**2.7 Privacy and data security**

2.7.1 Does the software comply with the European Union General Data Protection Regulation (EU GDPR)?*

🞅 Yes

🞅 No

Additional comment: …………………

2.7.2 Can multiple users accommodate the software with personal login and by secured password?*

🞅 Yes

🞅 No

🞅 Other: …………………

Additional comment: …………………

2.7.3 Is the database encoded?*

🞅 Yes

🞅 No

If yes, please specify the encoded method.

□ Password protected

□ Encrypted

□ Other: …………………

Additional comment: …………………

2.7.4 Are there data privacy method(s) used in data collection?*

🞅 Yes

🞅 No

🞅 Data collection is not possible in the software.

🞅 Other: …………………

If yes, please specify the data privacy method(s).

□ Informed consent

□ Data anonymization

□ Other: …………………

Additional comment: …………………

**2.8 Cost**

2.8.1 Is there a cost for an individual license? *

🞅 An individual license is free.

🞅 An individual license is subjected to a license fee per annum.

If applicable, please specify the individual license fee per annum.

Additional comment: …………………

2.8.2 Is there a cost for an institution license? *

🞅 An institution license is free.

🞅 An institution license is subjected to a license fee per annum.

If applicable, please specify the institution license fee per annum.

Additional comment: …………………

2.8.3 Is there a cost for an enterprise license? *

🞅 An enterprise license is free.

🞅 An enterprise license is subjected to a license fee per annum.

If applicable, please specify the enterprise license fee per annum.

Additional comment: …………………

2.8.4 Is there a cost for a maintenance and support contract? *

🞅 A maintenance and support contract is free.

🞅 A maintenance and support contract is subjected to a license fee per annum.

If applicable, please specify the maintenance and support contract fee per annum.

Additional comment: …………………

2.8.5 Is there a cost-effectiveness study of the software-based treatment in comparison with standard treatment available? *

🞅 Yes

🞅 No

If yes, please provide reference of the cost- effectiveness study.…………………

Additional comment: …………………

**Submission**

Thank you for your participation. Your answers will help us to evaluate your software accurately.

# Supplementary Tables and Figures

## Supplementary Table 1: Definition of evaluation grid

| **Criteria** | **Scoring** | **Definition** |
| --- | --- | --- |
|  |  |  |
| **1.User-friendliness and utilization** | | |
| 1.1 Supported platform(s) | 0  1 | one platform  more than one platform or web-based platform |
| 1.2 Availability of mobile application | 0  1 | no  yes or customized web-based version to mobile or tablet resolution |
| 1.3 Easy to download through internet | 0  1 | no  yes or web-based platform |
| 1.4 Easy to install | 0 to 1 | 0 = difficult, 1 = very easy or web-based platform |
| 1.5 Network installation | 0  1 | no  yes |
| 1.6 Multiple languages available | 0  1 | one language  more than one language |
| 1.7 Possibility of additional interface language requests | 0  1 | no  yes |
| 1.8 Availability of standalone version | 0  1 | no  yes |
| 1.9 Hospital information systems interfacing | 0  0.5  1 | no  under development  yes |
| 1.10 Manual entry of data | 0  1 | no  yes |
| 1.11 Data import from a remote central database | 0  1 | no  yes |
| 1.12 Data output and storage in a remote central database | 0  1 | no  yes |
| 1.13 Possibility of the data storage in the software | 0  1 | no  yes |
| 1.14 Possibility of database search | 0  1 | no  yes |
| 1.15 Possibility for each individual user to create their own database | 0  1 | no  yes |
| 1.16 Availability of add-on calculators (e.g. unit conversion, statistic calculation) | 0  1 | no  yes |
| 1.17 Global visual appeal | 0 to 1 | 0 = not visually appealing, 1 = visually appealing |
| 1.18 Customizable interface of the software | 0  1 | no  yes |
| 1.19 Wizard step-by-step interface in the software | 0  0.5  1 | no  help tab in the software  yes |
| 1.20 Easy to manually enter required data | 0  0 to 1  NA | User cannot manually input data.  0 = difficult, 1 = very easy  only available as connected version software |
| 1.21 Prior training requirement before using the software | 0  1 | prior training required  no prior training required |
| **2.User support** | | |
| 2.1 Availability of clinical manual | 0  1 | no  yes |
| 2.2 Availability of technical manual | 0  1 | no  yes |
| 2.3 User support | 0  0.25  0.5  0.75  1 | no  email response  help desk at specific time  help desk with live chat at specific time  help desk or live chat for 24 hours |
| 2.4 Availability of active community of software users (e.g. an online forum) | 0  1 | no  yes |
| 2.5 Availability of training for the users | 0  1 | no  yes |
| 2.6 Easy to update the software | 0  0.5  1 | Software update is not possible.  via the company website  Software is always updated (i.e. web-based software, in-house software). |
| **3. Computational aspect** | | |
| 3.1 Access to previous version | 0  1 | no  yes |
| 3.2 Availability of list of changes and bug fixes between versions | 0  1 | no  yes |
| 3.3 Access to source code by the user | 0  1 | no  yes |
| 3.4 Necessity of a runtime framework support | 0  1 | yes  no |
| 3.5 Minimum memory requirement to run the software | 0  1 | more than 4 GB RAM  not more than 4GB RAM |
| 3.6 Verbose bug outputs or message about error and warning condition | 0  1 | no  yes |
| 3.7 With graphic user interface | 0  1 | no  yes |
| 3.8 Capacity of structured data import | 0  1 | no  yes |
| 3.9 Capacity of structured data export | 0  1 | no  yes |
| **4. Population models** | | |
| 4.1 Number of drugs and drug classes | 0 to 1 | 0 = the least number of drugs and drug classes  1 = the most number of drug and drug classes |
| 4.2 Different populations | 0  0.25  0.5  0.75  1 | no population alternative  1 drugs with population alternatives  2 – 5 drugs with population alternatives  6 – 10 drugs with population alternatives  more than 10 drugs with population alternatives |
| 4.3 Model selection prior to implementing a model in the software | 0  1 | no  yes |
| 4.4 Automated model selection based on patients’ data | 0  1 | no  yes |
| 4.5 Availability of information on model selection to the user | 0  1 | no  yes |
| 4.6 Drug-specific references of the used model | 0  1 | no  yes |
| 4.7 Incorporation of models with inter-occasion variability | 0  1 | no  yes |
| 4.8 Non-Bayesian calculations | 0  1 | no  yes |
| 4.9 Bayesian analysis capabilities | 0  1 | no  yes |
| 4.10 A priori dosing regimen proposal | 0  1 | no  yes |
| 4.11 A priori loading dose proposal | 0  1 | no  yes |
| 4.12 Calculation and report of probability of target attainment | 0  1 | no  yes |
| 4.13 User-defined probability of target attainment | 0  1 | no  yes |
| 4.14 A posteriori dosing regimen proposal | 0  1 | no  yes |
| 4.15 A posteriori re-loading dose proposal | 0  1 | no  yes |
| 4.16 Number of drug measurements that can be entered | 0  1 | limited number of entered drug measurements  unlimited number of entered drug measurements |
| 4.17 Use of drug measurements with below and/or above the limit of quantifications | 0  1 | no  yes |
| 4.18 First dose handled with extrapolation to steady state | 0  1 | no  yes |
| 4.19 Non-steady state situations and/or irregular regimens handled | 0  0.5  1 | no  non-steady state situations or irregular regimens  both non-steady state situations and irregular regimens |
| 4.20 User-defined model | 0  1 | no  yes |
| 4.21 Parametrizable used covariates by the user | 0  1 | no  yes |
| 4.22 Model parameter values choosing by the user | 0  1 | no  yes |
| 4.23 Default boundaries of therapeutic target | 0  1 | no  yes |
| 4.24 User-defined boundaries of therapeutic target | 0  1 | no  yes |
| 4.25 Possibility to adjust/specify the dosage and obtain concentration simulation | 0  1 | no  yes |
| 4.26 Availability of optimal sampling time point module | 0  1 | no  yes |
| 4.27 Possibility of model refinement with collected data in the software | 0  1 | no  yes |
| **5. Quality and validation** | | |
| 5.1 Involvement of computer scientists | 0  1 | no  yes |
| 5.2 Involvement of computer engineers | 0  1 | no  yes |
| 5.3 Involvement of pharmacists | 0  1 | no  yes |
| 5.4 Involvement of clinicians | 0  1 | no  yes |
| 5.5 Involvement of pharmacometricians | 0  1 | no  yes |
| 5.6 Involvement of academic institutions | 0  1 | no  yes |
| 5.7 Involvement of pharmaceutical industry | 0  1 | no  yes |
| 5.8 Scientific publication describing the development of the software | 0  0.5  1 | no  abstract for a conference  yes |
| 5.9 Scientific publication describing the development of the models used in the software | 0  1 | no  yes |
| 5.10 Scientific publication describing a clinical research using the software | 0  1 | no  yes |
| 5.11 Model qualification prior to software implementation | 0  1 | no  yes |
| 5.12 Model performance evaluation with historical data drawn from records of the clinical setting in which the software is intended to be used. | 0  1 | no  yes |
| 5.13 Prospective validation of the selected model prior to implementing in the software | 0  1 | no  yes |
| 5.14 Verification of the software optimization algorithm against a well-established mathematical software (e.g. NONMEM, GNU scientific library) | 0  1 | no  yes |
| 5.15 Validation of data exchange when interfacing with another external system | 0  1 | no  yes |
| 5.16 Clinical validation of the software | 0  0.5  1 | no  part of ongoing study  yes |
| 5.17 Approval of the software as a software-based medical device | 0  1 | no  yes |
| 5.18 Continuous monitor of software performance | 0  1 | no  yes |
| **6. Output and report generation** | | |
| 6.1 A list of best dose candidate(s) suggestion | 0  1 | no  yes |
| 6.2 Comprehensive of dosing recommendation from the software | 0 to 1 | 0 = not easy to understand,  1 = straightforward and easy to understand |
| 6.3 Customizable dosing recommendation by the user | 0  1 | no  yes |
| 6.4 Report of individual PK parameters | 0  1 | no  yes |
| 6.5 Generation of PK | 0  1 | no  yes |
| 6.6 Prediction intervals and/or percentiles on the plot | 0  1 | no  yes |
| 6.7 Interactive adjustable plot | 0  1 | no  yes |
| 6.8 Global readability of the report  - not customizable report  - customizable report | 0  0 to 0.5  0.5 to 1 | no report  0 = difficult to read, 0.5 = easy to read  0.5 = difficult to read, 1 = easy to read |
| 6.9 Inclusion of the plot in the report | 0  1  NA | no  yes  no report |
| 6.10 Editable text field in the report | 0  1  NA | no  yes  no report |
| 6.11 Indication of user identity in the report | 0  1  NA | no  yes  no report |
| 6.12 Conversion of report format | 0  1  NA | no  yes  no report |
| **7. Privacy and data security** | | |
| 7.1 Compliance with the European Union General Data Protection Regulation (EU GDPR) or equivalent | 0  1 | no  yes |
| 7.2 Multiple user accommodation with personal login and by secured password | 0  1 | no  yes |
| 7.3 Encoded database | 0  1  NA | no encoded database  yes  Data cannot be store in the software. |
| 7.4 Confidentiality of data collection for model refinement in the software | 0  1  NA | no data privacy method apply  yes  Data collection is not possible in the software. |
| **8. Cost** | | |
| 8.1 Cost of an individual license per year | 0  1  NA | cost  free  An individual license is not available. |
| 8.2 Institution license at reduced cost | 0  1 | no  yes |
| 8.3 Cost of an institution license per year | 0  1  NA | cost  free  An institution license is not available. |
| 8.4 Cost of software version with additional functions | 0  1 | cost to access full version of the software  free or all users access to the same version. |
| 8.5 Cost for a maintenance and support contract | 0  1 | There is a cost for maintenance and support contract.  free or included in the license |
| 8.6 Cost-effectiveness analysis of the software | 0  0.5  1 | no  poster for a conference or part of ongoing study or white paper  scientific publication of cost-effectiveness analysis |

## Supplementary Table 2: Evaluation grid

|  | **Relative weight factor** | **Benchmarking score** | | | | | | | | | | |
| --- | --- | --- | --- | --- | --- | --- | --- | --- | --- | --- | --- | --- |
|  |  | **AutoKinetics** | **BestDose** | **DoseMeRx** | **ID-ODS** | **InsightRX Nova** | **MwPharm++** | **NextDose** | **PrecisePK** | **TDMx** | **TUCUXI** |  |
| 1.User-friendliness and utilization | | | | | | | | | | | | |
| 1.1 Supported platform(s) | 0.047 | 1 | 1 | 1 | 1 | 1 | 1 | 1 | 1 | 1 | 1 |  |
| 1.2 Availability of mobile application | 0.042 | 0 | 0 | 1 | 1 | 0 | 1 | 0 | 0 | 0 | 0 |  |
| 1.3 Easy to download through internet | 0.043 | 1 | 1 | 1 | 1 | 1 | 1 | 1 | 1 | 1 | 1 |  |
| 1.4 Easy to install | 0.057 | 0.1 | 1 | 1 | 1 | 1 | 0.7 | 1 | 1 | 1 | 0.55 |  |
| 1.5 Network installation | 0.046 | 1 | 1 | 1 | 0 | 1 | 1 | 1 | 1 | 1 | 0 |  |
| 1.6 Multiple languages available | 0.036 | 1 | 0 | 0 | 0 | 0 | 1 | 0 | 0 | 0 | 0 |  |
| 1.7 Possibility of additional interface language requests | 0.034 | 1 | 1 | 1 | 1 | 1 | 1 | 0 | 1 | 1 | 1 |  |
| 1.8 Availability of standalone version | 0.043 | 0 | 1 | 1 | 1 | 1 | 1 | 1 | 1 | 1 | 1 |  |
| 1.9 Hospital information systems interfacing | 0.059 | 1 | 0 | 1 | 0.5 | 1 | 1 | 0 | 1 | 0 | 1 |  |
| 1.10 Manual entry of data | 0.057 | 0 | 1 | 1 | 1 | 1 | 1 | 1 | 1 | 1 | 1 |  |
| 1.11 Data import from a remote central database | 0.058 | 1 | 0 | 1 | 0 | 1 | 1 | 0 | 1 | 0 | 1 |  |
| 1.12 Data output and storage in a remote central database | 0.057 | 1 | 0 | 1 | 0 | 1 | 1 | 0 | 1 | 0 | 1 |  |
| 1.13 Possibility of the data storage in the software | 0.054 | 1 | 1 | 1 | 1 | 1 | 1 | 1 | 1 | 0 | 0 |  |
| 1.14 Possibility of database search | 0.054 | 1 | 1 | 1 | 1 | 1 | 1 | 1 | 1 | 0 | 0 |  |
| 1.15 Possibility for each individual user to create their own database | 0.035 | 0 | 1 | 0 | 1 | 1 | 1 | 0 | 0 | 0 | 0 |  |
| 1.16 Availability of add-on calculators (e.g. unit conversion, statistic calculation) | 0.046 | 0 | 0 | 1 | 1 | 1 | 1 | 0 | 0 | 0 | 0 |  |
| 1.17 Global visual appeal | 0.053 | 0.48 | 0.63 | 0.75 | 0.53 | 0.83 | 0.33 | 0.68 | 0.80 | 0.63 | 0.60 |  |
| 1.18 Customizable interface of the software | 0.045 | 0 | 0 | 0 | 0 | 0 | 1 | 0 | 0 | 0 | 0 |  |
| 1.19 Wizard step-by-step interface in the software | 0.039 | 0 | 0 | 0.5 | 0 | 0.5 | 0.5 | 0.5 | 0 | 0 | 0 |  |
| 1.20 Easy to enter required data | 0.060 | NA | 0.48 | 0.85 | 0.70 | 0.85 | 0.38 | 0.68 | 0.80 | 0.70 | 0.63 |  |
| 1.21 Prior training requirement before using the software | 0.034 | 1 | 0 | 1 | 0 | 0 | 0 | 1 | 0 | 0 | 1 |  |
| **2.User support** | | | | | | | | | | | | |
| 2.1 Availability of clinical manual | 0.162 | 1 | 0 | 1 | 1 | 1 | 1 | 1 | 1 | 1 | 0 |  |
| 2.2 Availability of technical manual | 0.175 | 0 | 0 | 0 | 1 | 1 | 1 | 0 | 1 | 0 | 0 |  |
| 2.3 User support | 0.161 | 1 | 0.25 | 1 | 0.5 | 1 | 0.75 | 0.75 | 1 | 0.5 | 0.25 |  |
| 2.4 Availability of active community of software users (e.g. an online forum) | 0.138 | 0 | 1 | 0 | 0 | 1 | 0 | 0 | 0 | 0 | 0 |  |
| 2.5 Availability of training for the users | 0.184 | 1 | 1 | 1 | 1 | 1 | 1 | 0 | 1 | 1 | 0 |  |
| 2.6 Easy to update the software | 0.178 | 1 | 1 | 1 | 1 | 1 | 0.5 | 1 | 1 | 1 | 0.5 |  |
| **3.Computational aspect** | | | | | | | | | | | | |
| 3.1 Access to previous version | 0.109 | 1 | 0 | 0 | 0 | 0 | 1 | 0 | 0 | 0 | 0 |  |
| 3.2 Availability of list of changes and bug fixes between versions | 0.119 | 0 | 1 | 1 | 1 | 1 | 1 | 1 | 1 | 0 | 0 |  |
| 3.3 Access to source code by the user | 0.098 | 1 | 0 | 0 | 0 | 0 | 0 | 0 | 0 | 0 | 0 |  |
| 3.4 Necessity of a runtime framework support | 0.106 | 1 | 1 | 1 | 1 | 1 | 0 | 1 | 1 | 1 | 1 |  |
| 3.5 Minimum memory requirement to run the software | 0.096 | 1 | 1 | 1 | 1 | 1 | 1 | 1 | 1 | 1 | 1 |  |
| 3.6 Error and warning messages output to the user | 0.117 | 1 | 1 | 1 | 1 | 1 | 1 | 1 | 1 | 1 | 1 |  |
| 3.7 With graphic user interface | 0.125 | 1 | 1 | 1 | 1 | 1 | 1 | 1 | 1 | 1 | 1 |  |
| 3.8 Capacity of structured data import | 0.119 | 0 | 0 | 1 | 1 | 1 | 1 | 0 | 1 | 0 | 1 |  |
| 3.9 Capacity of structured data export | 0.111 | 1 | 0 | 1 | 1 | 1 | 1 | 1 | 1 | 0 | 1 |  |
| **4.Population models** | | | | | | | | | | | | |
| 4.1 Number of drugs | 0.040 | 0.13 | 0.19 | 0.75 | 0.38 | 0.75 | 1.00 | 0.44 | 0.56 | 0.13 | 0.31 |  |
| 4.2 Different population | 0.044 | 0 | 0.5 | 1 | 0.5 | 1 | 1 | 0.75 | 1 | 0.25 | 0 |  |
| 4.3 Model selection prior to implementing a model in the software | 0.042 | 1 | 1 | 1 | 1 | 1 | 1 | 1 | 1 | 1 | 0 |  |
| 4.4 Automated model selection based on patients’ data | 0.029 | 0 | 0 | 1 | 1 | 1 | 0 | 0 | 1 | 0 | 0 |  |
| 4.5 Availability of information on model selection to the user | 0.039 | 0 | 0 | 0 | 0 | 1 | 0 | 0 | 0 | 0 | 0 |  |
| 4.6 Drug-specific references of the used model | 0.041 | 1 | 1 | 1 | 1 | 1 | 1 | 1 | 1 | 1 | 1 |  |
| 4.7 Incorporation of models with inter-occasion variability | 0.036 | 0 | 0 | 0 | 0 | 1 | 1 | 1 | 1 | 0 | 1 |  |
| 4.8 Non-Bayesian calculations | 0.031 | 1 | 0 | 1 | 1 | 1 | 1 | 1 | 1 | 0 | 0 |  |
| 4.9 Bayesian analysis capabilities | 0.042 | 1 | 1 | 1 | 1 | 1 | 1 | 1 | 1 | 1 | 1 |  |
| 4.10 A priori dosing regimen proposal | 0.042 | 1 | 1 | 1 | 1 | 1 | 1 | 1 | 1 | 1 | 1 |  |
| 4.11 A priori loading dose proposal | 0.041 | 1 | 1 | 1 | 1 | 1 | 1 | 1 | 1 | 1 | 1 |  |
| 4.12 Calculation and report of probability of target attainment | 0.040 | 0 | 1 | 0 | 1 | 1 | 1 | 0 | 0 | 1 | 0 |  |
| 4.13 User-defined probability of target attainment | 0.038 | 0 | 0 | 0 | 0 | 0 | 0 | 0 | 0 | 0 | 0 |  |
| 4.14 A posteriori dosing regimen proposal | 0.044 | 1 | 1 | 1 | 1 | 1 | 1 | 1 | 1 | 1 | 1 |  |
| 4.15 A posteriori re-loading dose proposal | 0.036 | 1 | 1 | 1 | 1 | 1 | 1 | 0 | 1 | 1 | 1 |  |
| 4.16 Number of drug measurements that can be entered | 0.038 | 1 | 1 | 1 | 1 | 1 | 1 | 1 | 1 | 1 | 1 |  |
| 4.17 Use of drug measurements with below and above the limit of quantifications | 0.038 | 1 | 0 | 1 | 1 | 1 | 1 | 1 | 1 | 1 | 1 |  |
| 4.18 First dose handled with extrapolation to steady state | 0.032 | 1 | 1 | 1 | 1 | 1 | 1 | 1 | 1 | 1 | 1 |  |
| 4.19 Non-steady state situations and/or irregular regimens handled | 0.040 | 1 | 1 | 1 | 1 | 1 | 1 | 1 | 1 | 1 | 1 |  |
| 4.20 User-defined model | 0.032 | 0 | 0 | 0 | 0 | 0 | 1 | 0 | 1 | 1 | 1 |  |
| 4.21 Parametrizable used covariates by the user | 0.033 | 0 | 1 | 0 | 0 | 0 | 1 | 0 | 0 | 1 | 1 |  |
| 4.22 Model parameter values choosing by the user | 0.030 | 0 | 0 | 0 | 0 | 0 | 1 | 0 | 1 | 1 | 1 |  |
| 4.23 Default boundaries of therapeutic target | 0.032 | 1 | 0 | 1 | 1 | 1 | 1 | 1 | 0 | 1 | 1 |  |
| 4.24 User-defined boundaries of therapeutic target | 0.040 | 0 | 1 | 1 | 1 | 1 | 1 | 1 | 1 | 1 | 1 |  |
| 4.25 Possibility to adjust/specify the dosage and obtain concentration simulation | 0.038 | 0 | 1 | 1 | 1 | 1 | 1 | 1 | 1 | 1 | 1 |  |
| 4.26 Availability of optimal sampling time point module | 0.031 | 0 | 1 | 0 | 0 | 0 | 1 | 0 | 0 | 1 | 0 |  |
| 4.27 Possibility of model refinement with collected data in the software | 0.031 | 1 | 0 | 1 | 1 | 1 | 1 | 0 | 1 | 0 | 0 |  |
| **5.Quality and validation** | | | | | | | | | | | | |
| 5.1 Involvement of computer scientists | 0.055 | 1 | 1 | 1 | 0 | 1 | 1 | 0 | 1 | 1 | 1 |  |
| 5.2 Involvement of computer engineers | 0.054 | 1 | 1 | 1 | 1 | 1 | 1 | 0 | 1 | 0 | 1 |  |
| 5.3 Involvement of pharmacists | 0.055 | 1 | 1 | 1 | 1 | 1 | 1 | 0 | 1 | 1 | 1 |  |
| 5.4 Involvement of clinicians | 0.059 | 1 | 1 | 1 | 1 | 1 | 1 | 1 | 0 | 1 | 1 |  |
| 5.5 Involvement of pharmacometricians | 0.066 | 1 | 1 | 1 | 1 | 1 | 1 | 1 | 1 | 1 | 1 |  |
| 5.6 Involvement of academic institutions | 0.046 | 1 | 1 | 1 | 0 | 1 | 1 | 1 | 1 | 1 | 1 |  |
| 5.7 Involvement of pharmaceutical industry | 0.034 | 0 | 0 | 0 | 0 | 1 | 0 | 0 | 0 | 0 | 0 |  |
| 5.8 Scientific publication describing the development of the software | 0.045 | 0 | 1 | 0 | 1 | 0 | 1 | 0.5 | 1 | 1 | 1 |  |
| 5.9 Scientific publication describing the development of the models used in the software | 0.062 | 1 | 1 | 1 | 1 | 1 | 1 | 1 | 1 | 1 | 1 |  |
| 5.10 Scientific publication describing a clinical research using the software | 0.058 | 0 | 1 | 1 | 1 | 1 | 1 | 0 | 1 | 1 | 0 |  |
| 5.11 Model qualification prior to software implementation | 0.066 | 1 | 1 | 1 | 1 | 1 | 1 | 1 | 1 | 1 | 0 |  |
| 5.12 Model performance evaluation with historical data drawn from records of the clinical setting in which the software is intended to be used. | 0.059 | 1 | 1 | 1 | 1 | 1 | 1 | 1 | 1 | 1 | 0 |  |
| 5.13 Prospective validation of the selected model prior to implementing in the software | 0.060 | 1 | 1 | 1 | 1 | 1 | 1 | 0 | 1 | 0 | 0 |  |
| 5.14 Verification of the software optimization algorithm against a well-established mathematical software | 0.053 | 1 | 0 | 1 | 1 | 1 | 0 | 1 | 0 | 1 | 1 |  |
| 5.15 Validation of data exchange when interfacing with another external system | 0.055 | 1 | 0 | 1 | 0 | 1 | 1 | 0 | 1 | 0 | 0 |  |
| 5.16 Clinical validation of the software | 0.062 | 0.5 | 1 | 1 | 1 | 1 | 1 | 0 | 1 | 1 | 0 |  |
| 5.17 Approval of the software as a software-based medical device | 0.055 | 0 | 0 | 1 | 0 | 0 | 1 | 0 | 0 | 0 | 0 |  |
| 5.18 Continuous monitor of software performance | 0.056 | 1 | 0 | 1 | 0 | 1 | 1 | 1 | 1 | 0 | 0 |  |
| **6.Output and report generation** | | | | | | | | | | | | |
| 6.1 A list of best dose candidate(s) suggestion | 0.081 | 1 | 1 | 1 | 1 | 0 | 1 | 1 | 1 | 1 | 1 |  |
| 6.2 Comprehensive of dosing recommendation from the software | 0.093 | 0.70 | 0.58 | 0.65 | 0.68 | 0.73 | 0.53 | 0.75 | 0.85 | 0.65 | 0.60 |  |
| 6.3 Customizable dosing recommendation by the user | 0.082 | 0 | 1 | 1 | 0 | 1 | 1 | 1 | 1 | 1 | 1 |  |
| 6.4 Report of individual PK parameters | 0.085 | 1 | 1 | 1 | 1 | 1 | 1 | 1 | 1 | 1 | 1 |  |
| 6.5 Generation of PK plot | 0.084 | 1 | 1 | 1 | 1 | 1 | 1 | 1 | 1 | 1 | 1 |  |
| 6.6 Prediction intervals and/or percentiles on the plot | 0.075 | 1 | 1 | 0 | 1 | 1 | 1 | 0 | 0 | 1 | 1 |  |
| 6.7 Interactive adjustable plot | 0.078 | 0 | 0 | 1 | 0 | 1 | 0 | 1 | 1 | 0 | 1 |  |
| 6.8 Global readability of the report | 0.090 | 0 | 0 | 0.86 | 0.70 | 0.86 | 0.66 | 0.78 | 0.88 | 0 | 0.84 |  |
| 6.9 Inclusion of the plot in the report | 0.082 | NA | NA | 1 | 1 | 1 | 1 | 1 | 1 | NA | 1 |  |
| 6.10 Editable text field in the report | 0.080 | NA | NA | 1 | 0 | 1 | 0 | 1 | 1 | NA | 1 |  |
| 6.11 Indication of user identity in the report | 0.081 | NA | NA | 1 | 1 | 1 | 1 | 1 | 1 | NA | 1 |  |
| 6.12 Conversion of report format | 0.089 | NA | NA | 1 | 1 | 1 | 1 | 1 | 1 | NA | 1 |  |
| **7.Privacy and data security** | | | | | | | | | | | | |
| 7.1 Compliance with the European Union General Data Protection Regulation (EU GDPR) or equivalent | 0.264 | 1 | 0 | 1 | 0 | 1 | 1 | 1 | 1 | 1 | 1 |  |
| 7.2 Multiple user accommodation with personal login and by secured password | 0.251 | 1 | 1 | 1 | 1 | 1 | 1 | 1 | 1 | 0 | 0 |  |
| 7.3 Encoded database | 0.244 | 1 | 0 | 1 | 1 | 1 | 1 | 1 | 1 | NA | NA |  |
| 7.4 Confidentiality of data collection | 0.240 | 1 | NA | 1 | 1 | 1 | 1 | NA | 1 | NA | NA |  |
| **8.Cost** | | | | | | | | | | | | |
| 8.1 Cost of an individual license per year | 0.175 | 1 | 0 | NA | 1 | 0 | 0 | 1 | 0 | 1 | 1 |  |
| 8.2 Institution license at reduced cost | 0.175 | 1 | 1 | 1 | 1 | 1 | 1 | 1 | 1 | 1 | 1 |  |
| 8.3 Cost of an institution license per year | 0.180 | 1 | NA | 0 | 1 | 0 | 0 | 1 | 0 | 1 | 1 |  |
| 8.4 Cost of software version with additional functions | 0.165 | 1 | 1 | 0 | 1 | 0 | 1 | 1 | 0 | 1 | 1 |  |
| 8.5 Cost for a maintenance and support contract | 0.175 | 0 | 1 | 1 | 1 | 1 | 1 | 1 | 1 | 1 | 1 |  |
| 8.6 Cost-effectiveness analysis of the software | 0.130 | 0.5 | 1 | 0.5 | 0 | 0.5 | 0.5 | 0 | 0 | 0 | 0 |  |

##
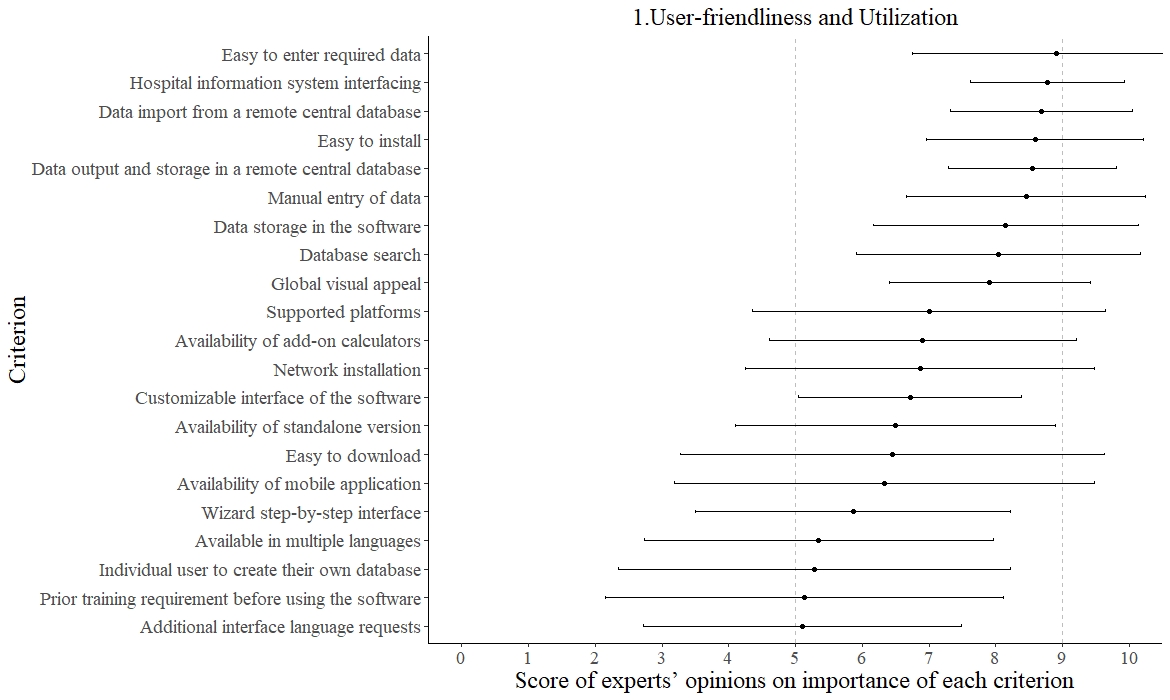
Supplementary Figure 1: Distributions of scores of experts’ opinions on each criterion in user-friendliness and utilization. Black solid dots represent average score and error bars represent ± 1standard deviation. Two gray vertical dashed lines at 5 and 9 represent limits of least important criteria and most important criteria, respectively.

##
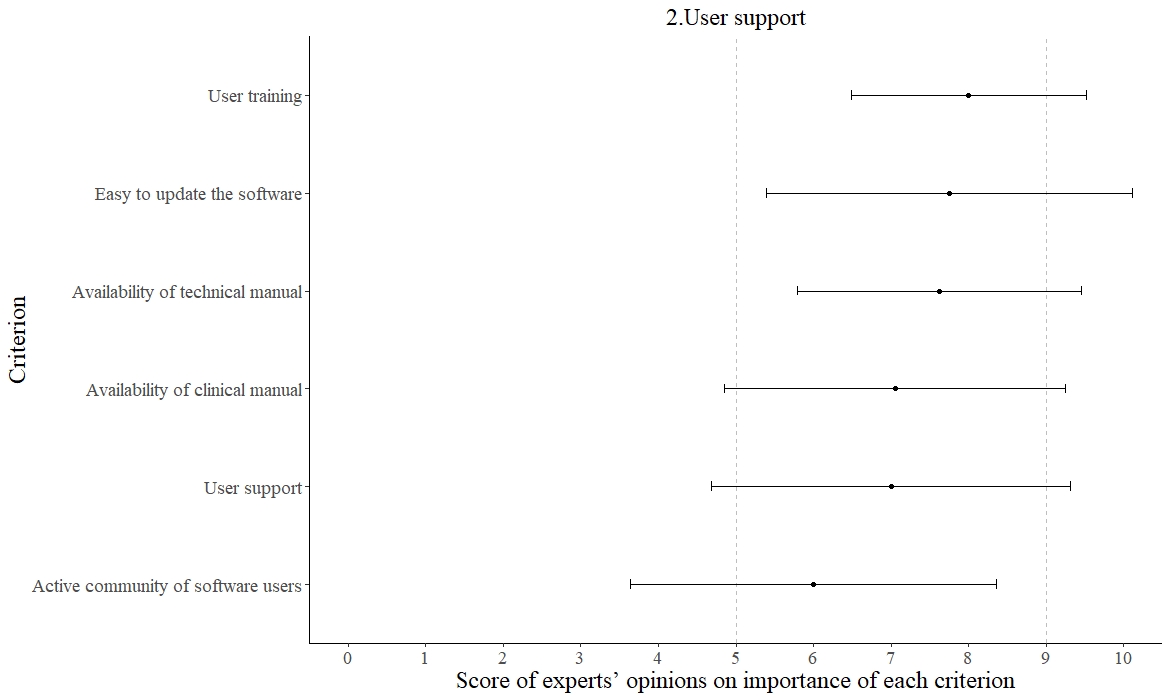
Supplementary Figure 2: Distributions of scores of experts’ opinions on each criterion in user support. Black solid dots represent average score and error bars represent ± 1standard deviation. Two gray vertical dashed lines at 5 and 9 represent limits of least important criteria and most important criteria, respectively.


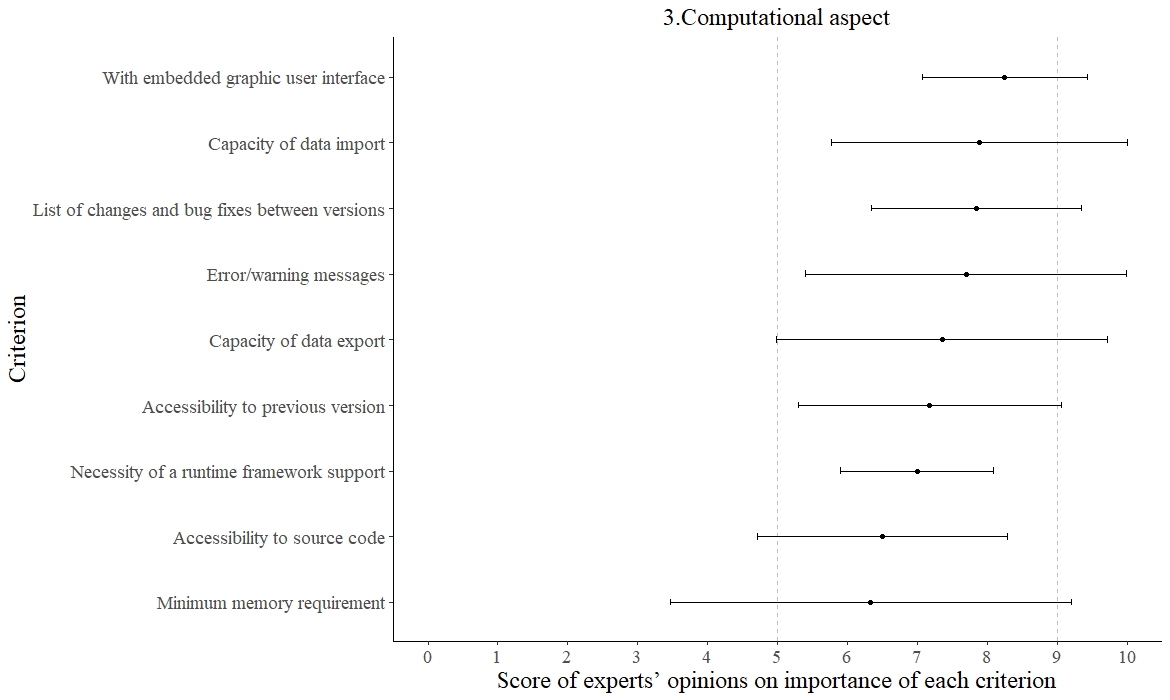


## Supplementary Figure 3: Distributions of scores of experts’ opinions on each criterion in computational aspect. Black solid dots represent average score and error bars represent ± 1standard deviation. Two gray vertical dashed lines at 5 and 9 represent limits of least important criteria and most important criteria, respectively.


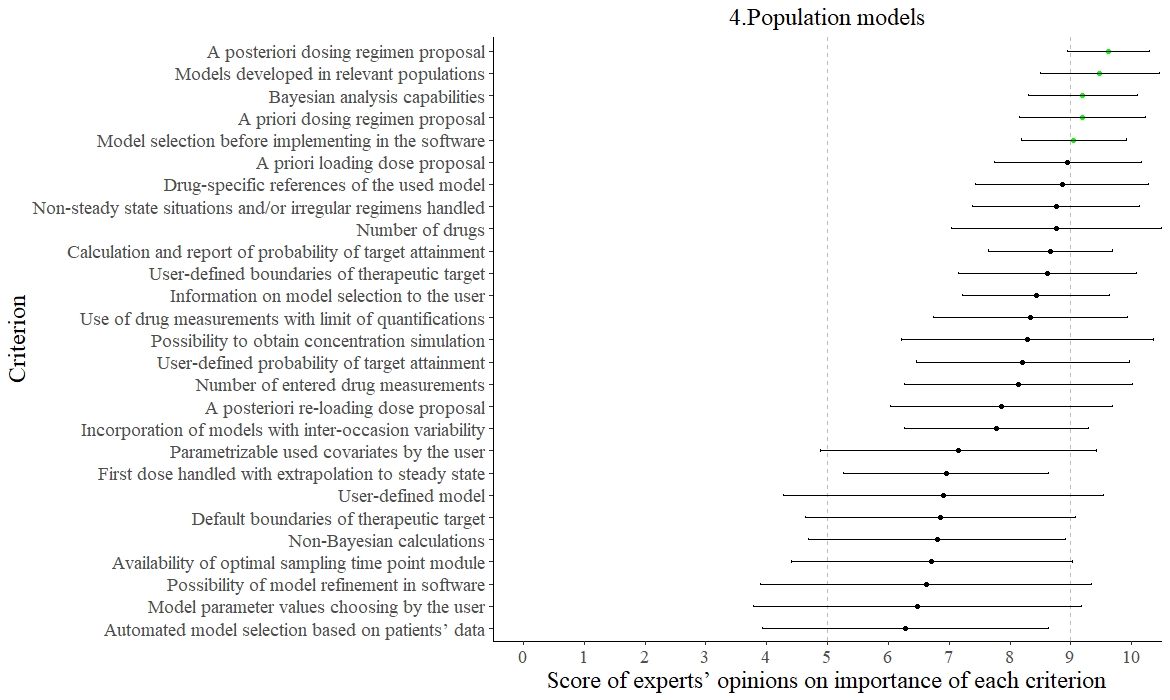


## Supplementary Figure 4: Distributions of scores of experts’ opinions on each criterion in population models. Black solid dots represent average score and error bars represent ± 1standard deviation. Two gray vertical dashed lines at 5 and 9 represent limits of least important criteria and most important criteria, respectively. Criteria with average score above 9 are presented in green solid dot.


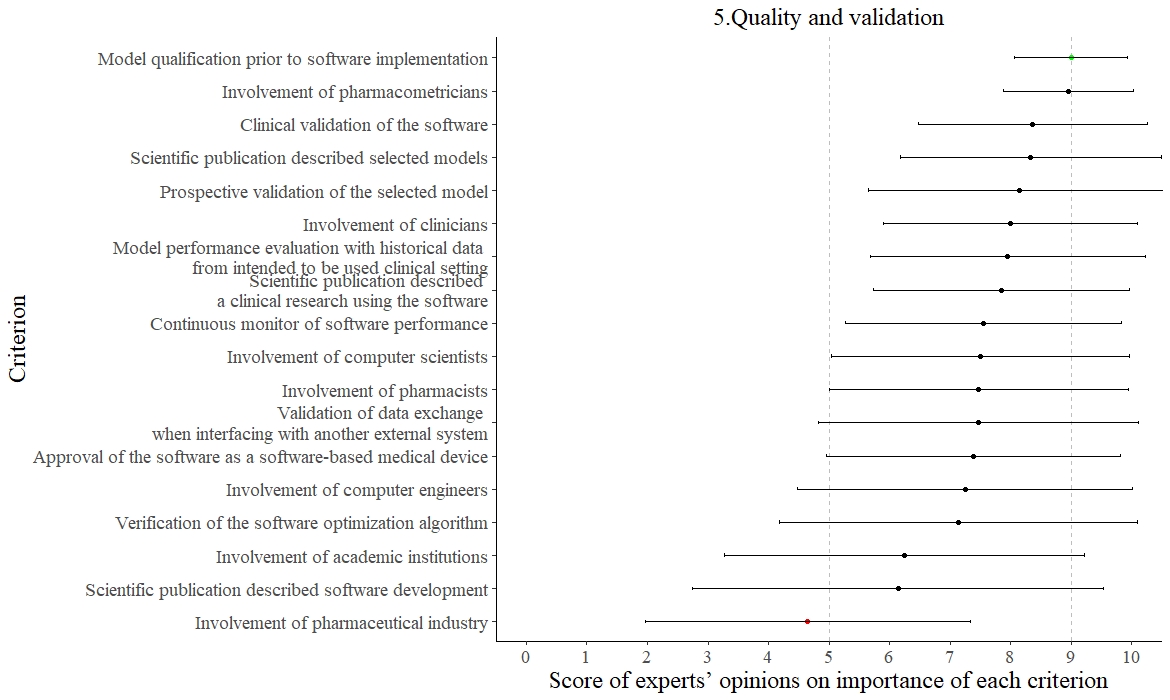


## Supplementary Figure 5: Distributions of scores of experts’ opinions on each criterion in quality and validation. Black solid dots represent average score and error bars represent ± 1standard deviation. Two gray vertical dashed lines at 5 and 9 represent limits of least important criteria and most important criteria, respectively. Criteria with average score below 5 are presented in red solid dot. Criteria with average score above 9 are presented in green solid dot.


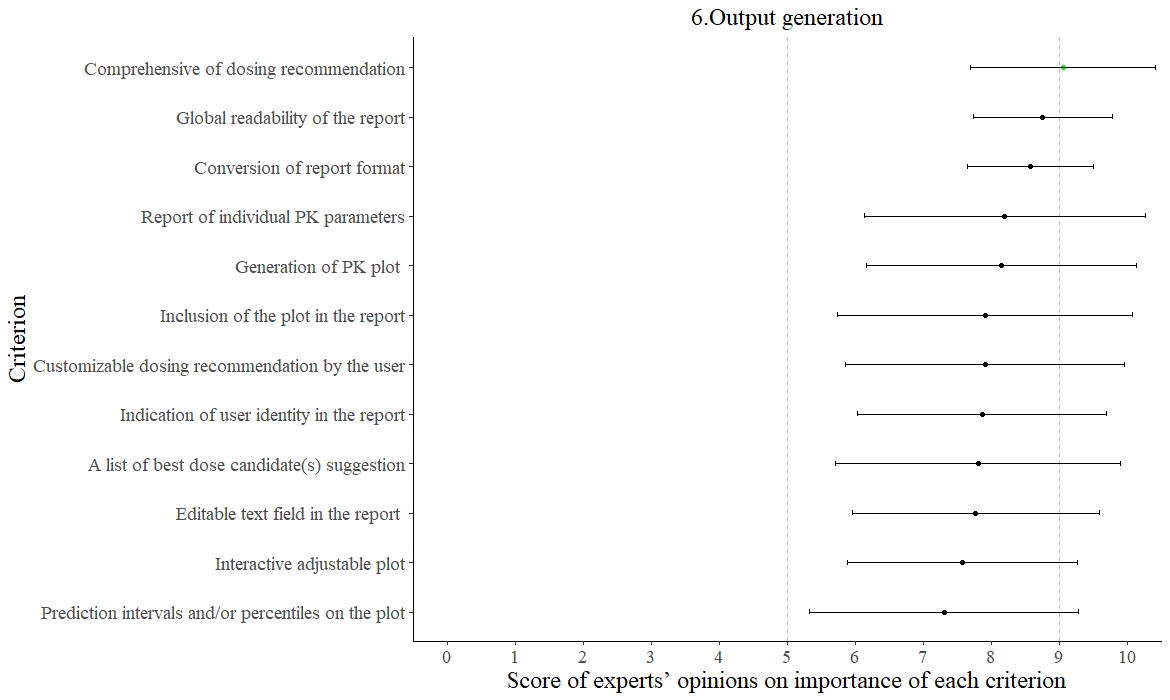


## Supplementary Figure 6: Distributions of scores of experts’ opinions on each criterion in output generation. Black solid dots represent average score and error bars represent ± 1standard deviation. Two gray vertical dashed lines at 5 and 9 represent limits of least important criteria and most important criteria, respectively. Criteria with average score above 9 are presented in green solid dot.


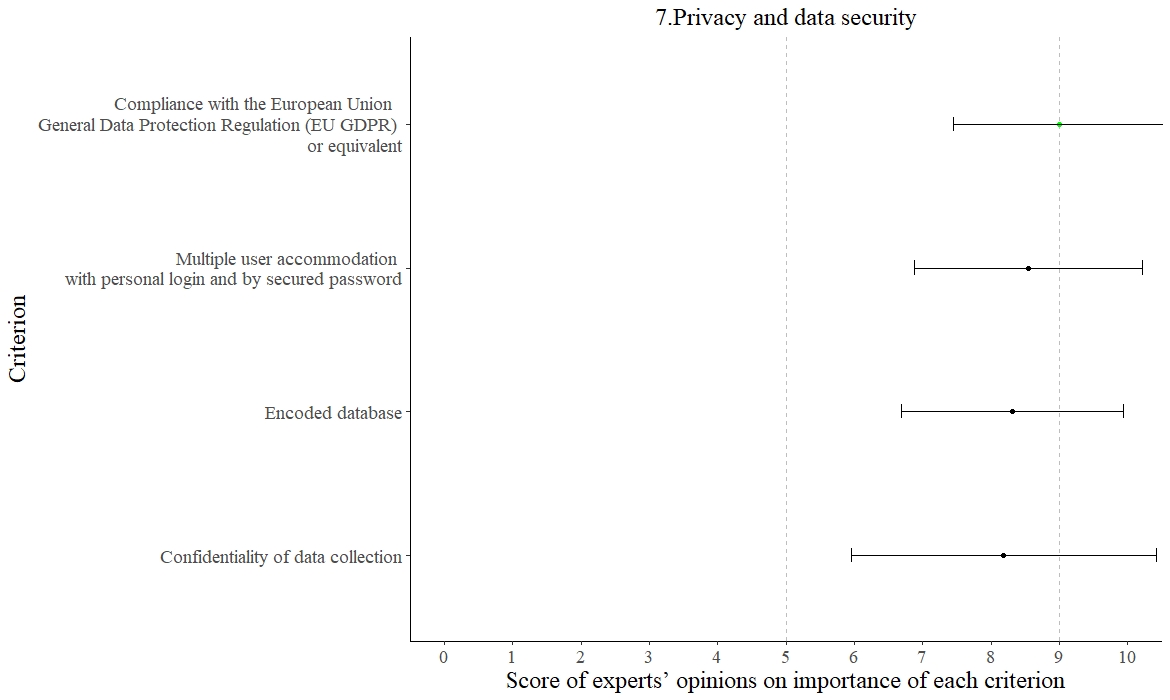


## Supplementary Figure 7: Distributions of scores of experts’ opinions on each criterion in privacy and data security. Black solid dots represent average score and error bars represent ± 1standard deviation. Two gray vertical dashed lines at 5 and 9 represent limits of least important criteria and most important criteria, respectively. Criteria with average score above 9 are presented in green solid dot.


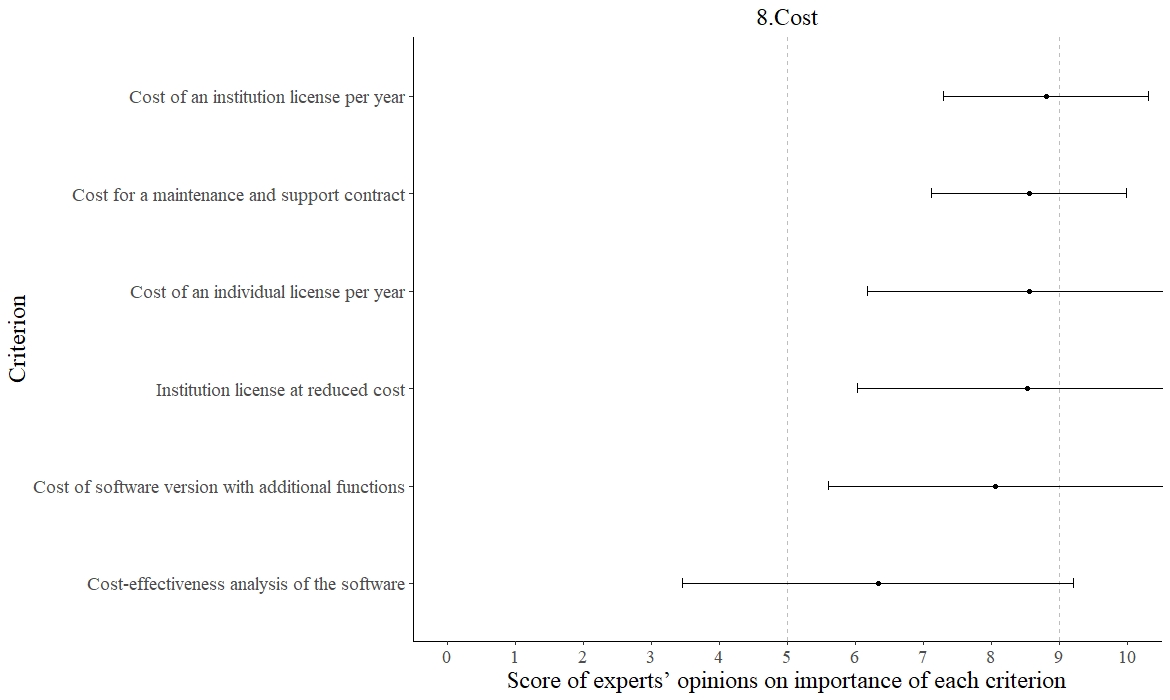


## Supplementary Figure 8: Distributions of scores of experts’ opinions on each criterion in cost. Black solid dots represent average score and error bars represent ± 1standard deviation. Two gray vertical dashed lines at 5 and 9 represent limits of least important criteria and most important criteria, respectively.
